# Mutual interaction of neurons and astrocytes derived from iPSCs with APP V717L mutation developed the astrocytic phenotypes of Alzheimer’s disease

**DOI:** 10.1186/s41232-023-00310-5

**Published:** 2024-02-28

**Authors:** Sopak Supakul, Rei Murakami, Chisato Oyama, Tomoko Shindo, Yuki Hatakeyama, Maika Itsuno, Hiroko Bannai, Shinsuke Shibata, Sumihiro Maeda, Hideyuki Okano

**Affiliations:** 1 Department of Physiology, Keio University School of Medicine, Tokyo, 160-8582 Japan; 2 Department of Electrical Engineering and Bioscience, School of Advanced Science and Engineering, Waseda University, Tokyo, 169-8555 Japan; 3 Electron Microscope Laboratory, Keio University School of Medicine, Tokyo, 160-8582 Japan; 4 Division of Microscopic Anatomy, Graduate School of Medical and Dental Sciences, Niigata University, Niigata, 951-8510 Japan

**Keywords:** Induced pluripotent stem cells (iPSCs), Neurons, Astrocytes, Co-culture model, Tripartite synapse, Alzheimer’s disease

## Abstract

**Background:**

The development of induced pluripotent stem cells (iPSCs) technology has enabled human cellular disease modeling for inaccessible cell types, such as neural cells in the brain. However, many of the iPSC-derived disease models established to date typically involve only a single cell type. These monoculture models are inadequate for accurately simulating the brain environment, where multiple cell types interact. The limited cell type diversity in monoculture models hinders the accurate recapitulation of disease phenotypes resulting from interactions between different cell types. Therefore, our goal was to create cell models that include multiple interacting cell types to better recapitulate disease phenotypes.

**Methods:**

To establish a co-culture model of neurons and astrocytes, we individually induced neurons and astrocytes from the same iPSCs using our novel differentiation methods, and then co-cultured them. We evaluated the effects of co-culture on neurons and astrocytes using immunocytochemistry, immuno-electron microscopy, and Ca^2+^ imaging. We also developed a co-culture model using iPSCs from a patient with familial Alzheimer's disease (AD) patient (*APP* ^V717L^ mutation) to investigate whether this model would manifest disease phenotypes not seen in the monoculture models.

**Results:**

The co-culture of the neurons and astrocytes increased the branching of astrocyte processes, the number of GFAP-positive cells, neuronal activities, the number of synapses, and the density of presynaptic vesicles. In addition, immuno-electron microscopy confirmed the formation of a tripartite synaptic structure in the co-culture model, and inhibition of glutamate transporters increased neuronal activity. Compared to the co-culture model of the control iPSCs, the co-culture model of familial AD developed astrogliosis-like phenotype, which was not observed in the monoculture model of astrocytes.

**Conclusions:**

Co-culture of iPSC-derived neurons and astrocytes enhanced the morphological changes mimicking the in vivo condition of both cell types. The formation of the functional tripartite synaptic structures in the co-culture model suggested the mutual interaction between the cells. Furthermore, the co-culture model with the *APP* ^V717L^ mutation expressed in neurons exhibited an astrocytic phenotype reminiscent of AD brain pathology. These results suggest that our co-culture model is a valuable tool for disease modeling of neurodegenerative diseases.

**Supplementary Information:**

The online version contains supplementary material available at 10.1186/s41232-023-00310-5.

## Background

Induced Pluripotent Stem Cells (iPSCs) are stem cells derived from adult somatic cells by introducing specific transcription factors such as *Oct3/4*, *Sox2*, *Klf4*, and *c-Myc* [1, 2]. Like embryonic stem cells (ESCs), the iPSCs have proliferative and pluripotent properties. Therefore, iPSCs can be differentiated into various cell types and generate human cell models in vitro [3]. Since the iPSCs hold the donor’s genetic information, it allows the in vitro study of human diseases with the patient’s own genetic background [4]. In recent years, especially for neurodegenerative disease research, the human cellular models generated from the iPSCs have been widely employed [5, 6]. For example, in the studies of Alzheimer’s disease (AD), the neurons induced from the iPSCs expressed the disease phenotypes according to the disease characteristic of the donor [7, 8].

Regarding the iPSC-derived cellular models used for disease analysis, the conventional approach involves the induction of iPSCs into a two-dimensional model (2D model) consisting of a single cell type. However, the monoculture model often lacks cell-cell interactions between different cell types of cells when compared to the human brain [9]. In recent years, the three-dimensional model (3D model) of brain organoids has been developed and applied to the study of neurodegenerative diseases including AD [10–13]. Although the brain organoid provides a platform for many cell types, it requires complicated culture procedures and relatively long culture periods. In particular, a relatively long culture period of more than 300 days is required to obtain specific cell types including astrocytes that exhibit astrocyte-like morphology [14]. Therefore, a model that promotes cell maturation and allows the study of cultured cells in a short period of time will be a useful tool for studying disease in a dish.

Furthermore, there was only a limited number of disease phenotypes that have been reported in a study of age-related diseases under the monoculture model of a single cell type. In particular, studies of AD have recapitulated only a few of the phenotypes of the non-neuronal population [15]. In recent years, a 2D cell model that includes more than a single cell type, called the co-culture model, has been developed. The co-culture model allows the analysis of the cell in a more physiological environment where more than one cell type is present compared to the monoculture model. A study of the co-culture model comprising the iPSC-derived neurons, astrocytes, and microglia succeeded in recapitulating neuroinflammation given the secreted complement component 3 (C3) from the microglia under the co-culture environment [16]. However, the changes in cellular properties, cell-cell interactions, and the phenotypes of other cell types under the co-culture system were still under investigation.

In the present study, our co-culture model of neurons and astrocytes which were separately differentiated from the iPSCs using the newly reported induction methods, was successfully established. This co-culture model demonstrated the promotion of morphological changes such as in vivo state in both neurons and astrocytes, the formation of functional tripartite synapse structure, and the enhancement of astrocytic disease phenotypes in the model of AD donor model (*APP* ^V717L^). Thus, this newly developed model provided a useful platform for modeling neurodegenerative diseases in human cells.

## Methods

### iPSC culture

The human iPSC lines used in this study are a healthy control line (201B7 line, female, 36 years old) [2] and a familial AD line with the *APP* ^V717L^ mutation (APP2E26 line, female, 57 years old) [17]. The cell culture method for iPSCs followed the protocol established by the Center for iPS Cell Research and Application (CiRA) [18]. The iPSCs were cultured in the feeder-free condition with StemFit® AK02N medium (Ajinomoto) on culture dishes pre-coated with 3.0 μg/mL iMatrix-511 silk (Matrixome 892021) in a humidified atmosphere with 5% CO_2_. The culture media were exchanged every other day. The iPSCs were cultured for 7 days before passaging using 0.5 × TrypLE Select (Thermo Fisher Scientific). After passage, cells were seeded onto a 6-well plate coated with 3.0 μg/mL iMatrix-511 silk at a density of 1.3 × 10^4^ cells/well in the presence of 10 μM Y-27632 (Nacalai).

### Neuron induction method

**Fig. 1:**
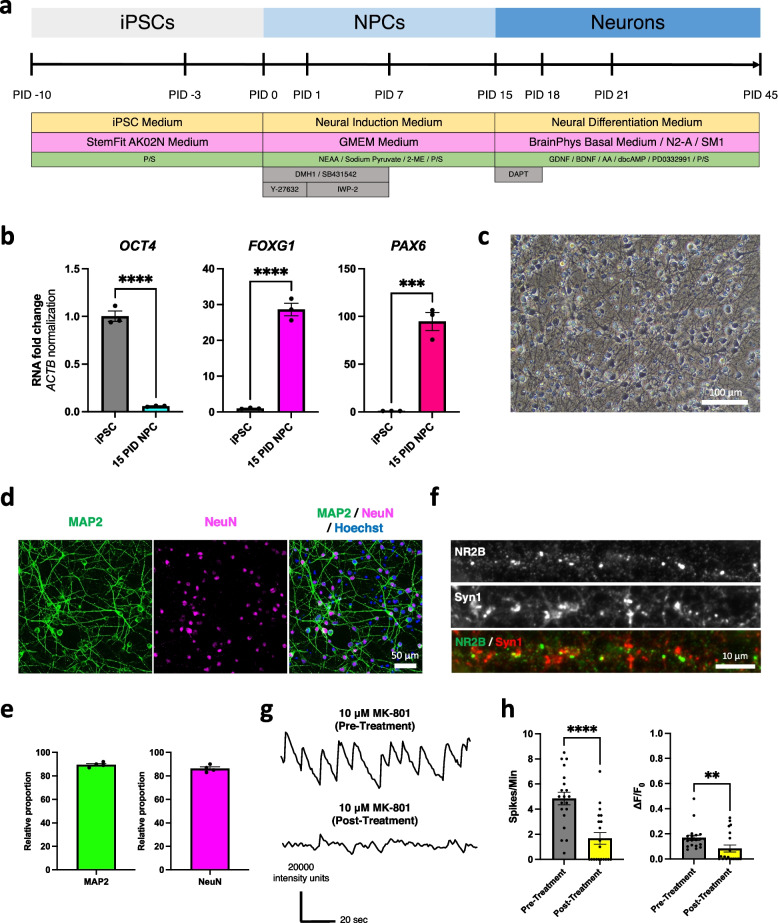
Generation and characterization of iPSC-derived neurons. **a** An induction method for the differentiation of neurons from iPSCs. **b** RT-qPCR analysis of stem cell marker (*OCT4*) and forebrain markers (*FOXG1* and *PAX6*) compared between the original iPSCs (*n* = 3) and the induced neural progenitor cells (NPCs) at PID 15 (*n* = 3) (three independent differentiations). Bars, mean ± SEM. *OCT4*: ****p < 0.0001; *FOXG1*: ****p < 0.0001; *PAX6*: ***p = 0.0006 (Unpaired t-test). **c** Representative bright-field image of iPSC-derived neurons at PID 45. Scale bar; 100 μm. **d** Immunocytochemistry images of iPSC-derived neurons at PID 45 stained with neuronal markers (MAP2 and NeuN). Scale bar; 50 μm. **e** Relative proportion of MAP2 and NeuN expression compared to Hoechst of the differentiated neurons (*n* = 4) (four independent differentiations). Bars, mean ± SEM. **f** Immunocytochemistry images of synaptic markers (NR2B and Syn1). Scale bar; 10 μm. **g** Representative waveforms of neuronal activity (Ca^2+^ oscillations) of iPSC-derived neurons at PID 45 measured by Ca imaging using Fluo-8 and the treatment with 10 μM MK-801 (N-Methyl-D-aspartate (NMDA) receptor blocker) compared with control. **h** Quantification of Ca^2+^ oscillations (frequency (Spikes/Min) and amplitude (ΔF/F_0_)) of iPSC-derived neurons at PID 45 measured by Ca imaging using Fluo-8 and the treatment with 10 μM MK-801 (*n* = 20) compared with the control (*n* = 20) (three independent experiments). Bars, mean ± SEM. ****p < 0.0001; **p = 0.0074 (Mann-Whitney test)

iPSCs were differentiated into neurons using the dual SMAD inhibition protocol previously reported [19, 20] (Fig. 1a). iPSCs were cultured in StemFit AK02N medium (Ajinomoto) supplemented with 0.5% Penicillin/Streptomycin (P/S) (Nacalai 09367-34). Prior to the induction of neural progenitor cells (NPCs), iPSCs were passaged onto a 6-well culture plate pre-coated with 3.0 μg/mL iMatrix-511 silk (Matrixome 892021) at a density of 40 × 10^4^ cells per well in 1.5 mL StemFit AK02N medium supplemented with 10 μM Y-27632 (Nacalai 08945-42). The culture medium was changed to fresh 4 mL StemFit AK02N everyday until the cells reached more than 80% confluence.

On the beginning of induction (PID 0), the culture medium was replaced with 4 mL of fresh neural induction medium. The neural induction medium consisted of GMEM medium (Wako 078-05525) supplemented with 8% KSR (Gibco 10828010), 0.1 mM non-essential amino acid solution (NEAA) (Nacalai 06344-56), 1 mM sodium pyruvate (Nacalai 06977-34), 0.1 mM 2-mercaptoethanol (2-ME) (Gibco 21985023), and 1% P/S (Nacalai 09367-34). To this medium, 2 μM DMH1 (Wako 041-33881), 2 μM SB431542 (Sigma-Aldrich), and 10 μM Y-27632 (Nacalai 08945-42) were added. From PID 1 to PID 7, the culture medium was changed every day to a fresh neural induction medium supplemented with 2 μM DMH1, 2 μM SB431542, and 2 μM IWP-2 (Sigma-Aldrich). From PID 7 to PID 15, the culture medium was replaced with a fresh neural induction medium without DMH1, SB431542, and IWP-2.

On PID 15, NPCs were dissociated using Accutase (Nacalai 12679-54) and re-plated onto a culture plate pre-coated with 1 μg/mL Poly-L-lysine solution (PLL) (Sigma-Aldrich P4832) and 4 ng/mL Laminin (R&D 3400-010-01) two days prior. The neural differentiation medium (BrainPhys basal medium/N2-A/SM1 kit (Stem Cell Technologies) supplemented with 10 ng/mL BDNF (R&D), 10 ng/mL GDNF (Alomone Labs G-240), 200 μM L-ascorbic acid (Sigma-Aldrich A4544), 0.5 mM dbcAMP (Nacalai 11540-61), 2 μM PD0332991 (Sigma-Aldrich PZ0199-5MG), 0.5% P/S (Nacalai 09367-34), 10 μM Y-27632, and 10 μM DAPT (Sigma-Aldrich D5942)) was used during the re-plating step. On PID 18, the medium was changed to a neural differentiation medium without Y-27632 and DAPT. After PID 18, half of the medium was replaced with a fresh neural differentiation medium every 3 days. The induced neurons were analyzed after PID 45.

### Astrocyte induction method

Astrocytes were induced from the feeder-free iPSCs using the previously reported astrocyte induction protocol [21]. Human iPSCs were cultured on mitomycin-C-treated SNL murine fibroblast feeder cells on 0.1% gelatin-coated culture dishes. Human iPSCs were cultured with human ES media at 37 ℃ in the humidified atmosphere of 3% CO_2_ until the beginning of induction. As ES media, Dulbeco's Modified Ealge Medium: Nutrient Mixture F-12 (DMEM/F-12 medium) (WAKO) was supplemented 20% KnockOut™ serum replacement (KSR) (Gibco), 1% 200 mM L-Glutamine (Gibco), 0.8% non-essential amino acids (NEAA) (Nacalai), 0.1 mM 2-mercaptoethanol (Sigma-Aldrich), 4 ng/mL fibroblast growth factor 2 (FGF2) (Pepro Tech), and Penicillin/Streptomycin (P/S). For the astrocyte differentiation, on PID 0, the iPSCs were removed from feeder cells with a dissociation solution (0.25% trypsin, 100 μg/mL collagenase IV (Invitrogen), 1 mM CaCl_2_, and 20% KSR) and cultured in FGF2-free human ES media with 10 μM Y-27632 to form Embryoid Bodies (EB) at 37 ℃ in a humidified atmosphere containing 5% CO_2_. To enhance the differentiation to neural linage, the media were changed EB media (DMEM/F12 medium containing 5% KSR, 1% L-Glutamine, 0.8% NEAA, 0.1 mM 2-mercaptoethanol, and Penicillin–Streptomycin) with 3 μM dorsomorphin (Sigma-Aldrich), 3 μM SB431542 (Sigma-Aldrich), 3 μM CHIR99021 (Focus Biomolecules) during PID 1 to 3. On PID 4, the media were changed to EB media supplemented with 1 μM retinoic acid (Sigma-Aldrich). The media were changed to EB media supplemented with 1 μM retinoic acid and 1 μM purmorphamine (Cayman) on PID 7, 10, and 13. On PID 16 the EBs were dissociated into single cells by TrypLE Select and dissociated cells were cultured with 1 ~ 4 × 10^4^ cells/mL cell density in the neurosphere media, which consisted of medium hormone mix (MHM) [22] supplemented with 2% B-27 (Nacalai), 0.8% NEAA, 1 μM purmorphamine, 20 ng/mL FGF2, and 10 ng/mL epidermal growth factor (EGF) (Pepro Tech) in a humidified atmosphere containing 5% CO_2_. The supplemented neurosphere media were changed to fresh media every 3 to 6 days to form neurospheres on PID 17 to 31. On PID 32 the primary neurospheres were dissociated with the same procedure as PID 16 and cultured with the same cell density in the neurosphere media containing 2% B-27, 0.8% NEAA, 20 ng/mL FGF2, and 10 ng/mL EGF in a humidified atmosphere containing 5% CO_2_. The supplemented neurosphere media were changed to fresh media every 3 to 6 days on PID 33 to 47. On PID 48 the secondary neurospheres were dissociated with the same procedure as PID 16 and 32, and plated 4 ~ 10 × 10^5^ cells/well in 6-well plates, pre-coated with 0.5% Matrigel (Corning) diluted by PBS, with iPSC-derived astrocytes differentiation media containing 2% B27, 0.8% NEAA, 10 ng/mL brain-derived neurotrophic factor (BDNF) (R&D), and 10 ng/mL glial cell line-derived neurotrophic factor (GDNF) (Alomone Labs G-240) in a humidified atmosphere containing 5% CO_2_ on PID 49 to 76. The media were changed to fresh media every 4 to 7 days until PID 77. On PID 77 the iPSC-derived astrocytes were detached using Accutase (Nacalai), and the cells were plated with 1 × 10^6^ cells/dish in 100-mm dishes pre-coated with 0.5% Matrigel in iPSC-derived astrocytes media in a humidified atmosphere containing 5% CO_2_. The plated cells were detached and plated with the same procedure as PID 77 once a week at least twice during this period on PID 78 to 97. On PID 98, iPSC-derived astrocytes were detached and re-plated for experiments and stored in serum-free CellBanker2 (Zenoaq) at -180 ℃.

### Establishment of the co-culture model

Neurons and astrocytes were induced separately from the same iPSC line. After removing DAPT from the neuronal assay on PID 18, the astrocytes were plated into the wells pre-plated with neurons at a ratio of 8:1 (neurons to astrocytes). After mixing neurons and astrocytes, cells were maintained using the neural differentiation medium. The cells were analyzed at 30 days after plating the astrocytes to the pre-plated neurons.

### Immunocytochemistry (ICC)

Cells were washed with PBS twice, then fixed with a 4% paraformaldehyde (PFA) solution (Wako 163-20145) for 15 min at room temperature, and subsequently washed three times with PBS. The cells were then permeabilized and blocked using a solution of 5% fetal bovine serum (FBS) and 0.3% Triton X-100 in PBS. Primary antibodies were applied to the cells and incubated overnight at 4 °C. On the following day, the cells were washed three times with PBS and incubated with secondary antibodies for 1 h at room temperature. Then, cell nuclei were stained with Hoechst 33258 (Dojindo Laboratories) diluted in PBS for 15 min. After two washes with PBS and one wash with MilliQ water, the cells were mounted using PermaFluor (Thermo Fisher Scientific TA-030-FM). Images of the cells were captured using a fluorescence microscope (BZ-X810; Keyence, Osaka, Japan and IX73; Olympus, Tokyo, Japan) or a confocal microscope (Zeiss LSM700; ZEISS Group, Oberkochen, Germany). The primary and secondary antibodies, along with their dilution ratios, are summarized in Supplementary Table 1. The PSD-95 intensity was quantified using MetaMorph software (Molecular Devices) as previously described [23].

### Reverse transcription-quantitative polymerase chain reaction (RT-qPCR)

RNA from the cultured cells was extracted using the RNeasy Mini Kit (Qiagen). Subsequently, cDNA was synthesized from the extracted RNA using the iScript cDNA synthesis kit (Bio-Rad). RT-qPCR was conducted utilizing 4 ng/μL cDNA, TB Green II, and Rox Dye II (TaKaRa), along with 20 μM of each primer. The amplification was carried out using a ViiA 7 Real-Time PCR System (Thermo Fisher Scientific 4453723) in accordance with the manufacturer's instructions. The detail of the primers used for qPCR was summarized in Supplementary Table 2.

### Glutamate reuptake assay

The L-Glutamate concentration was measured by the L-Glutamate measurement kit, YAMASA NEO (YAMASA). iPSC-derived astrocytes and iPSCs were plated at 1 × 10^5^ and 1 × 10^4^ cells/well on iMatrix pre-coated 96-well plate respectively. iPSC-derived astrocytes were cultured with iPSC-derived astrocyte media, and iPSCs were cultured with AK02N media for 7 days before starting the L-Glutamate concentration assay. L-Glutamate was dissolved in AK02N media at 150 mg/L and filtered through the 0.22 μm membrane (Millex®, Merck). For the experiment, iPSC-derived astrocytes and iPSCs were washed with PBS and replaced with AK02N media containing 150 mg/L L-Glutamate. L-Glutamate concentration in the media at 0, 1, 2, and 4 h after the experiment started was measured with the L-Glutamate measurement kit YAMASA NEO according to the manufacturer's protocol. The concentration of L-Glutamate at each time point was normalized by the value at 0 h.

### Ca^2+^ imaging

The co-culture model in a 96-well format at day 30 after mixing the cells was used for the Ca^2+^ imaging assay. Cells were incubated with 100 μL of neuronal induction medium, 2 μL of 500 μM Fluo-8 indicator (AAT Bioquest 21083), and 0.8 μL of 250 μM probenecid water (ThermoFisher Scientific P36400) at 37 °C for 15 min before imaging using an Olympus IX83 microscope. The cells were imaged 161 frames in a duration of 2 min and analyzed using the MetaMorph® Microscopy Automation and Image Analysis Software. Changes in Ca^2+^ oscillations of neurons in the co-culture model after the treatment with 20 μM inhibitors of Excitatory Amino Acid Transporter (EAAT) 1,2 (TFB-TBOA 2532) were measured to assess the function of tripartite synapse structure.

### Electron microscopy (EM)

Samples were prepared for EM observation as previously described [24, 25]. Briefly, the cells were plated onto the 4-well plastic chamber slide (Lab-Tek® Chamber Slide™ System 177437) with a density of neurons of 40 × 10^4^ cells and astrocytes of 5 × 10^4^ cells per well. Cells were fixed with 2.5% Glutaraldehyde (TAAB Laboratories, England, UK) in 0.1 M Cacodylate Buffer for 24 h at 4 °C. On the following day, after washing the cells using 0.1 M Cacodylate Buffer 3 times for 5 min each, the cells were fixed with 1% OsO_4_ (TAAB Laboratories, England, UK) in Cacodylate Buffer for 90 min at 4 °C. Cells were dehydrated in 50% Ethanol 2 times for 5 min each and stained with Uranium for 20 min at 4 °C. Then, cells were dehydrated in a series of 70–100% Ethanol and 100% Epon (27.0 g MNA, 51.3 g EPOK-812, 21.9 g DDSA, and 1.1 ml DMP-30 for 100 g Epon) (Oken-shoji Co. Ltd.) for 48 h at 4 °C. After incubating the cells in 100% Epon for 3 h at room temperature, cells were embedded and polymerized in 100% fresh Epon for 72 h at 60 °C. After polymerization, the cells were dissected from the slide glass at 100 °C, fixed on the sectioning stage for 24 h at 60 °C, and dried up in the desiccator for 24 h at room temperature. Ultrathin sections of the cells with a thickness of 80 nm were prepared using an ultramicrotome (Leica UC7; Leica Biosystems, Wetzlar, Germany) with a diamond knife (Ultra, DiATOME). After 24 h of incubation at room temperature, the ultrathin sections were stained with uranyl acetate and lead citrate respectively at room temperature. The sections were examined and imaged using a Transmission Electron Microscope (TEM) (JEM-1400plus; JEOL, Tokyo, Japan) at 100 keV.

For pre-embedding immuno-EM (iEM), cells in the 4-well plastic chamber slide (Lab-Tek® Chamber Slide™ System 177437) were fixed with 4% PFA in 0.1 M Phosphate Buffer (PB, pH 7.4) (Muto Pure Chemicals) for 30 min at 4 °C, washed three times for 3 min each in PB, and blocked in a blocking solution (5% Block Ace (KAC) and 0.01% saponin in 0.1 M PB) for 1 h at room temperature. Cells were then incubated with a mouse anti-S100β antibody (1:250) (Supplementary Table 1) in a blocking solution for 72 h at 4 °C. After washing washed ten times for 10 min each in 0.1 M PB with 0.005% saponin, cells were incubated with Alexa Fluor 488 / FluoroNanogold-conjugated goat anti-mouse secondary antibody (1:100) (Thermo Fisher Scientific) and Hoechst 33258 (1:1000) (Sigma-Aldrich) for 24 h at 4 °C. After washing washed ten times for 10 min each in 0.1 M PB with 0.005% saponin, areas of interest were identified utilizing Alexa Fluor 488 signals under a Zeiss Axio Imager M1 Fluorescence Microscope (ZEISS Group, Oberkochen, Germany). The sections were fixed with 2.5% Glutaraldehyde in 0.1 M PB for 1 h at 4 °C, washed with 0.1 M PB three times for 5 min each, washed with 50 mM HEPES (pH 5.8) (Sigma-Aldrich) three times for 10 min each, and washed with diluted water for 1 min. Next, the nanogold signals were enhanced using silver enhancement solution for 2 min and 30 s at 25 °C. After washing with diluted water five times for 1 min each, the cells were fixed with 1% osmium tetroxide (TAAB Laboratories) for 1.5 h at 4 °C, washed with distilled water, and dehydrated with 50% Ethanol. The cells were *en bloc* stained with 2% Uranium in 50% Ethanol for 20 min at 4 °C. After, samples were dehydrated in a series of 70–100% Ethanol, incubated in 100% Epon (Oken-shoji Co. Ltd.) for 30 min at room temperature followed by several hours at 4 °C, and polymerized in 100% Epon for 72 h at 60 °C. The resin blocks of the samples were trimmed and ultrathin sectioned (thickness of 80 nm) with an ultramicrotome (Leica UC7; Leica Biosystems, Wetzlar, Germany). After 24 h of incubation at room temperature, the sections were stained with uranyl acetate and lead citrate for 10 min. Samples were imaged using TEM (JEM-1400plus; JEOL, Tokyo, Japan) at 100 keV.

### Glutamate toxicity assay

The co-culture model and neuronal monoculture model at day 30 after mixing the cells were treated with 15 μg/mL L-Glutamate (Wako) for one week. To investigate cell viability, the cells were treated with 1 μg/mL Calcein-AM (DOJINDO) in the culture media for 1 h, washed with PBS, and fixed with 4% paraformaldehyde for 16 h at 4 ℃. Then, fluorescent signals of Calcein-AM were detected by In Cell Analyzer 6000 (Cytiva, Marlborough, United States). Calcein-positive and MAP2-positive neurons were counted by In Cell Developer (Cytiva, Marlborough, United States). The surviving neuron ratios were calculated as Calcein-positive and MAP2-positive neurons/Hoechst.

### DNA sequencing and genotyping

Genomic DNA from the cells was extracted using the DNeasy Blood and Tissue kit (Qiagen). The extracted DNA was subjected to PCR amplification using primers for the fragment of *APP* (Supplementary Table 2). The Proflex PCR system (Thermo Fisher Scientific) was used with the following cycling parameters: an initial denaturation at 95 °C for 2 min, followed by 35 cycles of denaturation at 95 °C for 30 s, annealing at 58 °C for 30 s, and extension at 72 °C for 1 min. Gel electrophoresis was performed on each PCR product using 125 mV for 30 min. The DNA band corresponding to the target product size was excised, and the DNA was purified using the QIAquick Gel Extraction Kit following the manufacturer's instructions. Purified DNA (20 ng) and 1 μL of each 10 μM primer were used for Sanger sequencing conducted by Eurofins Genomics K.K., Japan.

### Enzyme-linked immunosorbent assay (ELISA)

ELISA was conducted using the conditioned media from both mono-cultured and co-cultured cells to quantify the levels of secreted amyloid-beta (Aβ)_1–42_ and Aβ_1-40._ The assay was conducted following the manufacturer's instructions with the ELISA kit (Wako 298-64601; Wako 296-64401). Briefly, the Standard Solution was prepared by diluting Aβ peptides with Standard Diluent at several concentrations. Then, 100 μL of the Standard Solution and the conditioned medium was applied to an antibody-coated plate, the plate was sealed and incubated at 4 °C overnight. On the next day, the Standard Solution and the conditioned medium were removed from the wells, and the wells were washed 4–5 times with a washing solution diluted in 1xPBS. Subsequently, 100 μL/well of the Horseradish peroxidase (HRP) antibody solution was added to the wells, and the plate was sealed and incubated at 4 °C (1 h for Aβ_1-42_ and 2 h for Aβ_1-40_). After the incubation, the HRP antibodies were removed, and the wells were washed 4–5 times with the washing solution. Then, 100 μL/well of 3,3', 5,5;-tetramethylbenzidine (TMB) solution was added, and the plate was placed in a dark environment at room temperature for 30 min. Later, without removing the TMB solution, 100 μL/well of the Stop Solution was added to the wells. The absorbance of the conditioned media from each Aβ_1-42_ and Aβ_1-40_ plate was measured at 450 nm using the iMarkTM Microplate Reader (Bio-Rad). Subsequently, the Aβ_1-42_ levels, Aβ_1-40_ levels, and Aβ_1-42_/ Aβ_1-40_ ratios were calculated.

### Statistical analysis

Statistical analysis was performed with GraphPad Prism (Version 9.4.1 (458); GraphPad Software, Boston, MA, USA). All data are presented as the mean ± SEM. The Unpaired t-test and Mann-Whitney test were used for comparison between the 2 groups depending on the normality assessed by the Shapiro-Wilk test. One-way ANOVA and Kruskal-Wallis test were used for group analysis upon the normality test by the Shapiro–Wilk test. Two-way ANOVA was used for the glutamate reuptake and Sholl analyses. Differences were considered significant at *p < 0.05, **p < 0.01, ***p < 0.001, and ****p < 0.0001.

## Results

### Human iPSCs were induced into the functional neurons

To induce the functional neurons, the iPSCs were differentiated into neurons by the dual SMAD inhibition using small compounds via neural progenitor cells (NPCs) as previously described [19, 20] (Fig. 1a). The qPCR of the induced NPCs at PID 15 confirmed the decreased expression of a pluripotent stem cell marker (*OCT4*) and increased expression of the cortical forebrain markers (*FOXG1* and *PAX6*) compared to the original iPSCs (Fig. 1b). The induced neurons at PID 45 have normal morphology (Fig. 1c), and the neuronal markers (MAP2 and NeuN) and synaptic markers (NR2B and Syn1) were expressed (Fig. 1d, f). The iPSCs were differentiated into neurons with high efficacy using our method (Fig. 1e). To assess the function of the induced neurons, Ca^2+^ imaging using the Fluo-8 indicator was performed on the neurons at PID 45. In addition to the neuronal activities observed in the induced neurons, the treatment with 10 μM MK-801 (blocker of N-Methyl-D-aspartate (NMDA) receptor) resulted in decreased Ca^2+^ oscillations (Fig. 1g, h). Thus, this confirmed the NMDA receptor-dependent activities of the iPSCs-derived neurons.

### Human iPSCs were induced into functional astrocytes

**Fig. 2:**
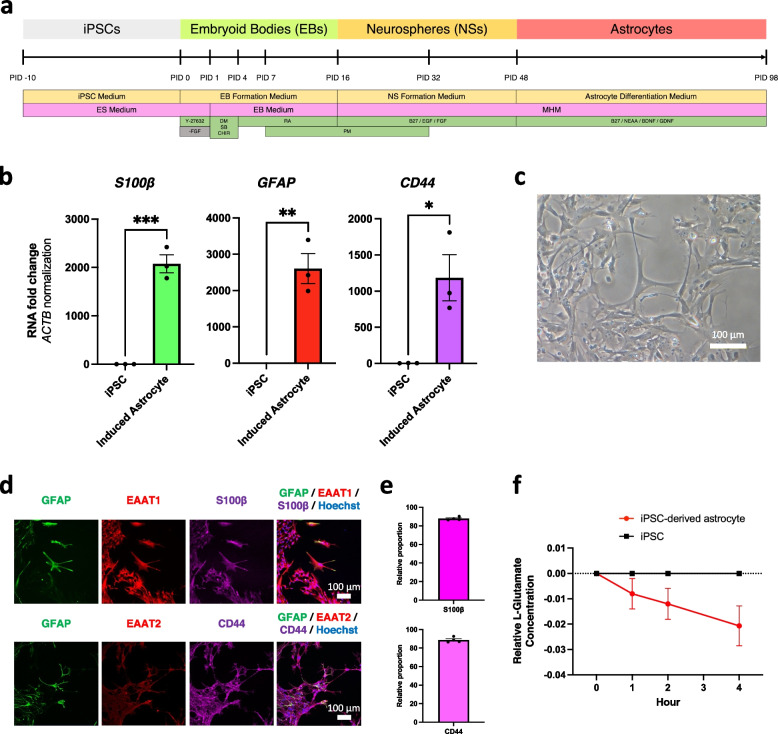
Generation of iPSC-derived astrocytes. **a** Induction method for the differentiation of astrocytes from iPSCs. **b** RT-qPCR analysis of astrocyte markers (*S100β*, *GFAP*, *CD44*) compared between the original iPSCs (*n* = 3) and the induced astrocytes (*n* = 3) (three independent differentiations). Bars, mean ± SEM. *S100β*: ***p = 0.0004; *GFAP*: **p = 0.0033; *CD44*: *p = 0.0208 (Unpaired t-test). **c** Representative bright-field image of iPSC-derived astrocytes. Scale bar; 100 μm. **d** Immunocytochemistry images of iPSC-derived astrocytes (GFAP, EAAT1, EAAT2, S100β, and CD44). Scale bar; 100 μm. **e** Relative proportion of S100β and CD44 expression compared to Hoechst of the differentiated astrocytes (*n* = 4) (four independent differentiations). Bars, mean ± SEM.** f** Glutamate reuptake assay for iPSC-derived astrocytes measured at zero, one, two, and four hours after adding 150 mg/L L-Glutamate (*n* = 3). Bars, mean ± SEM. *p = 0.0433 (Dunnett’s test following a Two-way ANOVA)

iPSCs were differentiated into astrocytes via the formation of embryoid bodies (EBs) and neurospheres (NSs) according to the previous report [21] (Fig. 2a). This induction method allows the derivation of a relatively pure astrocyte population through the passages of the cells several times after the induction of NSs without exposure to serum. The qPCR results of the iPSC-derived astrocytes showed significantly increased expression levels of *S100β*, *GFAP*, and *CD44* compared to the original iPSCs at mRNA levels (Fig. 2b). The differentiated cells showed characteristic morphology mimicking astrocyte morphology (Fig. 2c). The immunocytochemistry also showed that the induced astrocytes expressed GFAP, EAAT1, EAAT2, S100β, and CD44 (Fig. 2d), which are astrocytic markers. S100β and CD44 were expressed in high efficacy using our method (Fig. 2e). As a functional assay, the L-Glutamate uptake assay was conducted. Conditioned media of iPSC-derived astrocytes and iPSCs were sampled time-dependently. The concentration of L-Glutamate in the conditioned media of the induced astrocytes decreased in a time-dependent manner, while there were no changes in the concentration of L-Glutamate in the conditioned media of the iPSCs even after 4 h (Fig. 2f). These results suggested that the iPSCs were successfully differentiated into functional astrocytes.

### Co-culturing of neurons and astrocytes induced cellular changes resembling the astrocytes in vivo

**Fig. 3:**
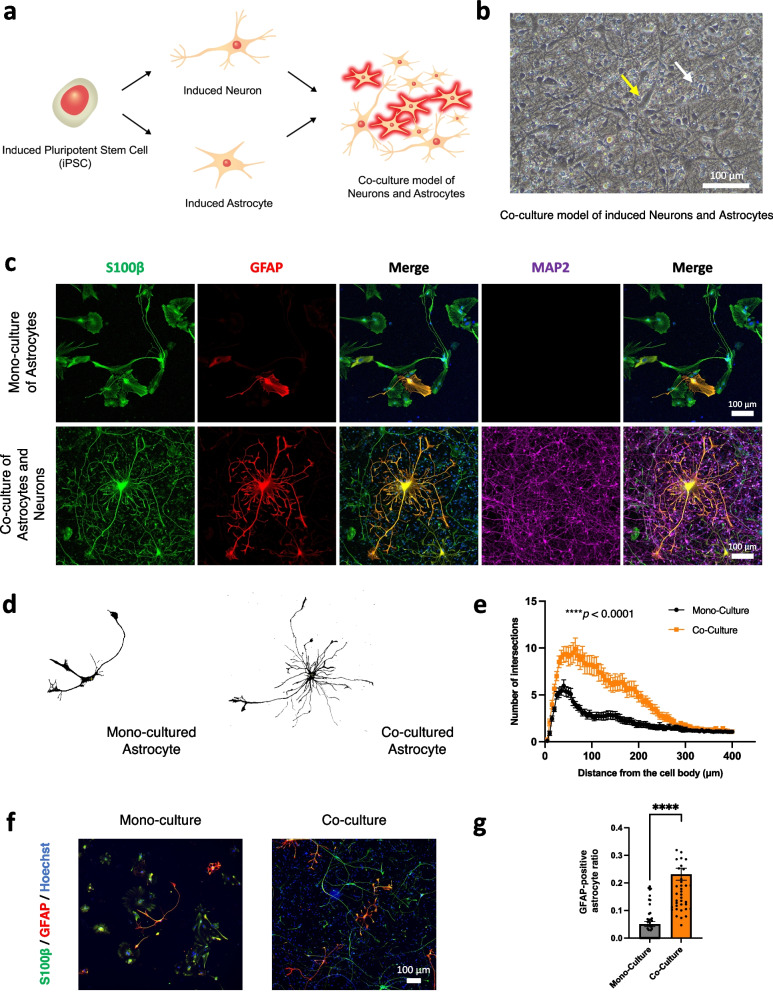
Establishment of iPSC-derived neurons and astrocytes co-culture system and cellular changes of the iPSC-derived astrocytes in the co-culture system. **a** Schematic experimental paradigm of the co-culture using individually induced neurons and astrocytes from the same iPSCs. **b** Bright-field images of the co-culture model of the iPSC-derived neurons (white arrow) and astrocytes (yellow arrow) at 30 days after co-culturing of the cells. Scale bar; 100 μm. **c** Representative immunocytochemistry images of the iPSC-derived mono-culture model of astrocytes and co-culture model, at 30 days after co-culturing of the cells, stained with antibodies specific for S100β, GFAP, and MAP2. Scale bar; 100 μm. **d** Representative images of iPSC-derived astrocytes in the mono- and co-culture models. **e** Sholl analysis showing the complexity of the iPSC-derived astrocyte of the mono-culture model (*n* = 30) and co-culture model (*n* = 30) (three independent experiments). Bars, mean ± SEM. ****p < 0.0001 (Two-way ANOVA). **f** Immunocytochemistry images of iPSC-derived astrocytes stained with S100β and GFAP in the mono- and co-culture models. Scale bar; 100 μm. **g** Quantification and comparison of the GFAP/S100β ratios between the mono-culture model (*n* = 45) and the co-culture model (*n* = 45). ****p < 0.0001 (Mann-Whitney test)

To enable the mutual interaction between neurons and astrocytes, we aimed to establish a novel co-culture system of the iPSC-derived neurons and astrocytes. The neurons and astrocytes were individually induced from the identical iPSCs as described above. Then, the iPSC-derived astrocytes were plated onto the wells pre-plated with iPSC-derived neurons in the neural differentiation medium to establish the co-culture model (Fig. 3a). Bright-field images of the co-culture model revealed the co-existence of two different types of cells even 30 days after co-culturing neurons and astrocytes. The induced astrocytes have larger soma, long and thick cell processes (in the yellow arrow of Fig. 3b), while the induced neurons have smaller cell soma (in the white arrow of Fig. 3b). Immunocytochemistry images of the co-culture model revealed the increased number of cell processes of the induced astrocytes (GFAP-positive cell) (Fig. 3c).

To further confirm the morphological changes in astrocytes, we carried out a Sholl analysis of the cells. Sholl analysis revealed the increased complexity, as measured by the increase in the number of intersections at a distance from the center of the cells, of the induced astrocytes in the co-culture model (Fig. 3d, e). Furthermore, to examine if the induced astrocytes would increase their activation when co-culturing with the neurons, we stained the cells with GFAP. As a result, immunocytochemistry showed that astrocytes in the co-culture model had a higher ratio of GFAP/S100β compared to astrocytes in the monoculture model (Fig. 3f, g). These results suggested that the co-culture with neurons could enhance morphological changes and activation of the iPSC-derived astrocytes.

### Neurons in the co-culture model were more mature compared to neurons in the monoculture model

**Fig. 4:**
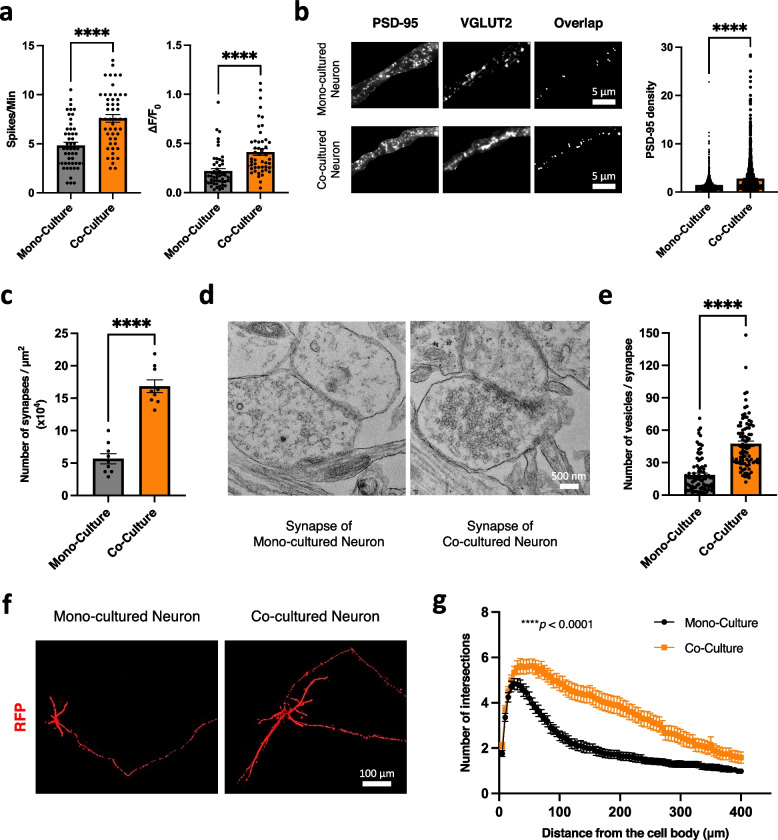
Cellular changes of iPSC-derived neurons in the co-culture system. **a** Ca^2+^ oscillations (frequency (Spikes/Min) and amplitude (ΔF/F_0_)) of the neurons measured by Ca.^2+^ imaging using the Fluo-8 indicator in the mono-culture model (*n* = 50) and co-culture model (*n* = 50) (three independent experiments). ****p < 0.0001 (Unpaired t-test). **b** PSD-95 staining and the quantification of PSD-95 density (PSD-95 intensity/PSD-95 dot after overlapping with VGLUT2) of the iPSC-derived neurons in the mono-culture model (*n* = 1,468) and co-culture model (*n* = 1,242) (three independent experiments). Scale bar; 5 μm. Bars, mean ± SEM. ****p < 0.0001 (Mann-Whitney test). **c** Quantification of the number of synapses between mono-culture (*n* = 9) and co-culture models (*n* = 9) (three independent experiments). Bars, mean ± SEM. ****p < 0.0001 (Unpaired t-test). **d** Representative transmission electron microscopy (TEM) images of synaptic structures in the mono-culture model and in the co-culture model. Scale bar; 500 nm. **e** Quantification of the number of synaptic vesicles in the presynaptic terminal of neurons in co-culture models (*n* = 90) compared to the mono-culture models (*n* = 90) (three independent experiments). Bars, mean ± SEM. ****p < 0.0001 (Mann-Whitney test). **f** Representative images of neurite branching of the iPSC-derived neurons in the mono- and co-culture model labeled with pCl-DsRed protein and supplementary stained with anti-RFP antibody. Scale bar; 100 μm. **g** Sholl analysis showing the complexity of neurites of the iPSC-derived neurons in the mono-culture model (*n* = 50) and co-culture model (*n* = 50) (three independent experiments). Bars, mean ± SEM. ****p < 0.0001 (Two-way ANOVA)

To investigate changes in neurons, we compared the activity and the morphology of neurons in the co-culture model with neurons in the monoculture model. First, Ca^2+^ imaging using the Fluo-8 indicator revealed increased neuronal activity in the neurons co-cultured with astrocytes (Fig. 4a) (Supplementary Video 1). The immunocytochemistry of PSD-95, the post-synaptic marker, revealed a higher density of the post-synaptic compartment in the co-culture model compared to the monoculture model (Fig. 4b). In addition, quantification of the number of synapses on transmission electron microscopy (TEM) showed that there were more synapses in the co-culture model than the monoculture model of neurons (Fig. 4c). The TEM images of the synapse of the neurons also revealed the pre-synaptic compartments fulfilled with more presynaptic vesicles and a thicker post-synaptic density structure in the post-synaptic compartments of the neurons in the co-culture model (Fig. 4d, e). These results suggested the enhanced maturation of iPSC-derived neurons in the co-culture model compared to those in the monoculture model.

To further investigate the morphological changes of the neurons in the co-culture and monoculture, the induced neurons were labeled by introducing the pCl-dsRed plasmid to the cells and double-labeled with an anti-RFP antibody to supplement the intrinsic fluorescent signals of RFP (Fig. 4f). Compared to the monoculture model, neurons in the co-culture model showed increased neurite branching and complexity (Fig. 4g). Together, these results indicated that both the function and the structure of the induced neurons were more mature in the co-culture system with astrocytes compared to those of the neurons in the monoculture system.

### The co-culture improves neuronal viability compared to the monoculture under a stressed condition

**Fig. 5:**
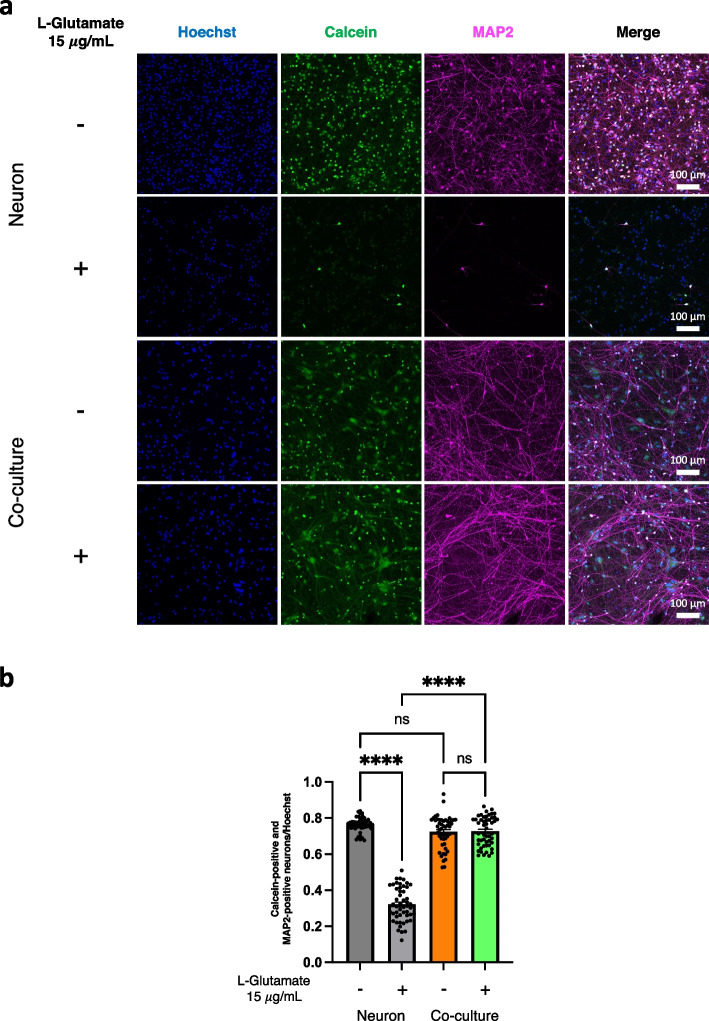
Improving neuronal vulnerability against excessive L-Glutamate treatment by co-culturing with astrocytes. **a** Representative immunocytochemistry images of the co-culture model and neuronal mono-culture with or without excessive L-Glutamate treatment (15 μg/mL) for one week stained with Calcein-AM reagent and an antibody specific for MAP2. Scale bar; 100 μm. **b** Quantification and comparison of surviving ratio of iPSC-derived neurons (Calcein-positive and MAP2-positive neurons/Hoechst) among the mono-culture model of neurons and the co-culture model with and without excessive L-Glutamate treatment (15 μg/mL) for one week (*n* = 50 each) (three independent experiments). Bars, mean ± SEM. ****p < 0.0001 (One-way ANOVA)

To investigate whether astrocytes would protect neurons from neuronal cell death induced by neurotoxins [26], we examined the glutamate toxicity assay (15 μg/mL L-Glutamate treatment for one week) followed by the staining with Calcein-AM. The surviving neuron ratios (Calcein-positive and MAP2-positive neurons/Hoechst) were not different between the monoculture model and the co-culture model when not treated with excessive L-Glutamate. Although the monoculture model of neurons with excessive L-Glutamate treatment showed a lower surviving neuron ratio compared to the monoculture models without excessive amount of L-Glutamate, the surviving ratio of the neurons in the co-culture model with excessive L-Glutamate treatment was not changed (Fig. 5a, b). This indicated that astrocytes in the co-culture model not only enhanced neuronal maturation but also protected neurons from excessive amount of glutamate.

### Functional tripartite synapse formation in the co-culture model of neurons and astrocytes

**Fig. 6:**
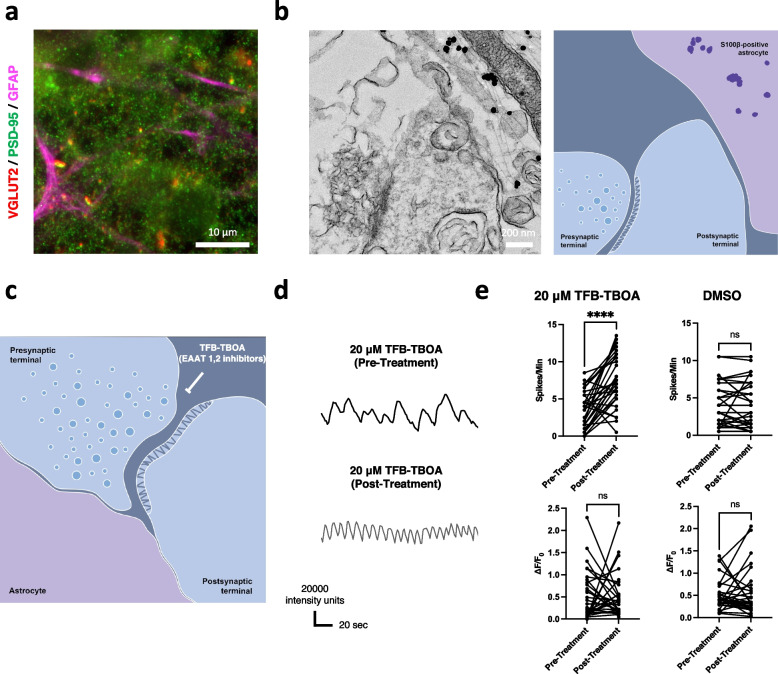
Tripartite synapse structure in the co-culture system and the cell-cell interaction between the induced neurons and astrocytes. **a** Immunocytochemistry image showing the neuronal synaptic markers (VGLUT2 and PSD95) and the astrocyte marker (GFAP). Scale bar; 10 μm. **b** Immuno-electron microscopy (iEM) image showing the neuronal synaptic terminals, and astrocytic process stained with S100β. Scale bar; 200 nm. **c** Evaluation of neuronal excitability measured by Ca imaging using Fluo-8 indicator in the co-culture model after treatment with 20 μM TFB-TBOA (Excitatory Amino Acid Transporter (EAAT) 1,2 inhibitor). **d** Representative waveforms of neuronal activity (Ca^2+^ oscillations) of the co-culture model before and after treatment with 20 μM TFB-TBOA. **e** Quantification of neuronal firing (Spikes/Min and ΔF/F_0_) of the co-culture model after treatment with 20 μM TFB-TBOA (*n* = 30) compared to the treatment with DMSO (n = 30) (three independent experiments). ****p < 0.0001 (Mann-Whitney test)

We examined whether the co-culture model of the induced neurons and astrocytes from the iPSCs recapitulate the tripartite synapse structure comprised the presynaptic and postsynaptic terminals of neurons and the attaching process of the astrocytes [27]. First, the immunocytochemistry of pre-, post-synaptic markers (VGLUT2 and PSD95), and the astrocyte marker (GFAP) in the co-culture model visualized the close associations of them (Fig. 6a). Further imaging utilizing the immuno-electron microscope (iEM) revealed the neuronal synapse structure and the associated process of astrocyte stained with S100β (Fig. 6b). Treatment with 20 μM TFB-TBOA (Excitatory Amino Acid Transporter (EAAT) 1,2 inhibitor) to the co-culture model increased Ca^2+^ oscillations (Spikes/Min) of the neurons (Fig. 6c-e) (Supplementary Video 2). Thus, this suggested functional tripartite synapse formation in our co-culture model.

### Co-culturing developed AD-like phenotypes of astrocytes induced from the iPSCs with familial AD mutation (*APP *^V717L^)

**Fig. 7:**
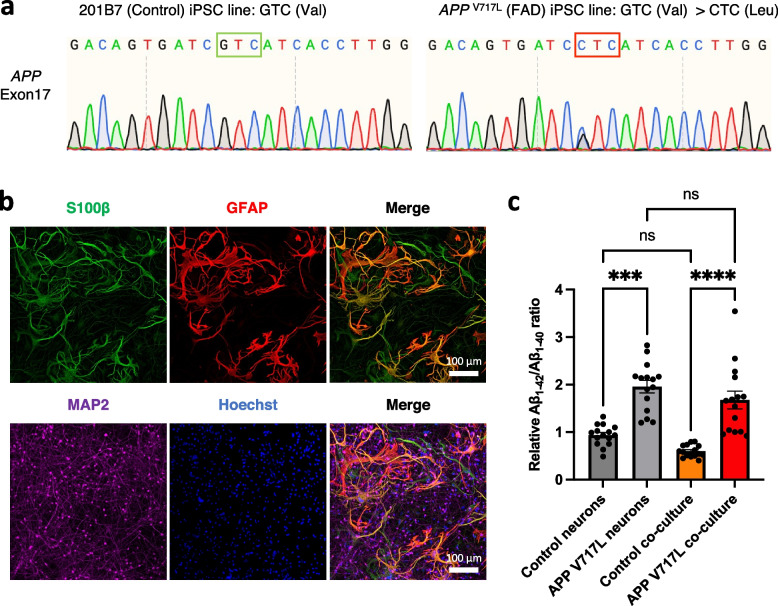
Establishment of the co-culture model from the iPSCs of the familial AD donor with *APP* ^V717L^ mutation (APP2E26 line). **a** Genotyping results of the APP2E26 iPSCs showing the G > C point mutation at *APP* Exon 17 (*APP *^V717L^). **b** Representative immunocytochemistry images of the co-culture model derived from the APP2E26 iPSC line stained with antibodies specific for S100β, GFAP, and MAP2. Scale bar; 100 μm. **c** Relative Aβ_1-42_/Aβ_1-40_ ratio of the iPSC-derived neurons and co-culture model of control 201B7 line (neurons (*n* = 15); co-culture (*n* = 15)) and fAD APP2E26 line (neurons (*n* = 15); co-culture (*n* = 15)) (three independent experiments). Bars, mean ± SEM. ***p < 0.001; ****p < 0.0001 (Kruskal-Wallis test)

To examine if our co-culture model can recapitulate the AD-like phenotypes, we co-cultured neurons and astrocytes derived from iPSCs bearing the familial AD (fAD) mutation (*APP* ^V717L^) [17, 28]. The iPSC line (APP2E26 line) from a female donor harboring the mutation of familial AD (*APP* ^V717L^) were obtained [17], and we confirmed a single nucleotide substitution (G > C) at *APP* Exon 17 (Fig. 7a) [29]. We co-cultured the neurons and astrocytes from the fAD donor (*APP* ^V717L^) and confirmed them by immunostaining of the markers (Fig. 7b). ELISA measurement of Aβ_1-42_ and Aβ_1-40_ confirmed the increased ratio of Aβ_1-42_/Aβ_1-40_ in the *APP* ^V717L^ co-culture model compared to the control co-culture model as previously reported [17, 28] (Fig. 7c). The *APP* ^V717L^ iPSCs can be differentiated into both neurons and astrocytes with high efficacy (Supplementary Fig. 1a-d). The astrocytes differentiated from the *APP* ^V717L^ iPSCs also demonstrated the glutamate reuptake function. Then, there is no significant difference between the astrocyte of *APP* ^V717L^ and the control (Supplementary Fig. 1e). Furthermore, after co-culture, an increase in astrocytic complexity and an increase in neurite branching were compatible in the control and *APP* ^V717L^. This indicated that both control and *APP* ^V717L^ neurons and astrocytes enhanced astrocytic and neuronal maturation, respectively, to a comparable extent (Supplementary Fig. 1f, g).

**Fig. 8:**
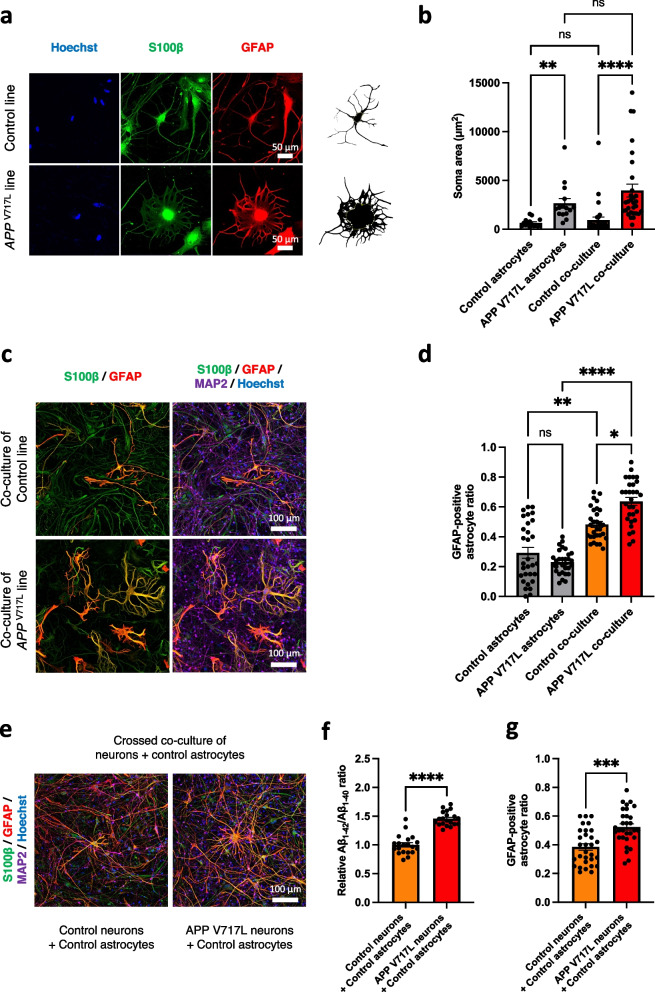
Astrocytic phenotypes of AD (*APP* ^V717L^ mutation) developed in the co-culture model of iPSC-derived neurons and astrocytes. **a** Representative immunocytochemistry (S100β and GFAP) images of iPSC-derived astrocytes in the co-culture model derived from the control and familial AD iPSC lines. Scale bar; 50 μm. **b** Quantification and comparison of soma areas (μm^2^) among the control astrocytes (*n* = 15), *APP* ^V717L^ astrocytes (*n* = 15), control co-culture model (*n* = 30), and *APP* ^V717L^ co-culture model (*n* = 30) (three independent experiments). Bars, mean ± SEM. *p < 0.05 (Kruskal-Wallis test). **c** Representative immunocytochemistry images of the control (201B7 line) and familial AD (*APP* ^V717L^ line) iPSC-derived astrocytes in the co-culture stained with antibodies specific for S100β and GFAP. Scale bar; 100 μm. **d** Quantification of the GFAP/S100β ratio among the control astrocytes (*n* = 30), the APP V717L astrocytes (*n* = 30), control co-culture model (*n* = 30), and APP V717L co-culture model (*n* = 30) (three independent experiments). Bars, mean ± SEM. *p < 0.05; ** *p* < 0.01; ****p < 0.0001 (Kruskal-Wallis test). **e** Representative immunocytochemistry images of the co-culture model of control (201B7 line) iPSC-derived astrocytes with the control (201B7 line) or familial AD (the *APP* ^V717L^ line) iPSC-derived neurons. Scale bar; 100 μm. **f** Relative Aβ_1-42_/Aβ_1-40_ ratio of the co-culture models of control (201B7 line) iPSC-derived astrocytes with the control (201B7 line) (*n* = 20) or the familial AD (the *APP* ^V717L^ line) iPSC-derived neurons (n = 20). Bars, mean ± SEM. ****p < 0.0001 (Unpaired-t test). **g** Quantification of the GFAP/S100β ratio of the co-culture model of the control (201B7 line) iPSC-derived astrocytes with the control (201B7 line) (*n* = 30) or the familial AD (the *APP* ^V717L^ line) iPSC-derived neurons (*n* = 30) (three independent experiments). Bars, mean ± SEM. ***p = 0.0002 (Mann-Whitney test)

Since APP is mainly expressed in neurons but not in astrocytes, we hypothesized that our co-culture method developed AD-like phenotypes of astrocytes derived from fAD iPSCs that are hindered in monoculture. The astrocytes in the *APP* ^V717L^ co-culture model showed larger cell bodies and shorter cell processes, which resembled astrocytic hypertrophy found in brains of AD patients and other neurodegenerative diseases [30] (Fig. 8a). The quantification of the GFAP-positive astrocytes revealed that soma areas of astrocytes in the *APP* ^V717L^ were significantly larger compared to the astrocytes in the control in both monoculture model and co-culture model (Fig. 8b). In addition, the co-culture model of the *APP* ^V717L^ donor increased the GFAP/S100β ratio compared to the co-culture model of a healthy control donor (Fig. 8c). However, the GFAP/S100β ratio was not altered between the monoculture models of healthy control and *APP* ^V717L^ astrocytes alone (Fig. 8d).

To further investigate the mechanism of the development of astrocytic phenotypes, we performed the crossed co-culture of the control astrocytes with *APP* ^V717L^ neurons (Fig. 8e). The increase in the Aβ_1-42_/Aβ_1-40_ ratio in the co-culture model of the control astrocytes and the *APP* ^V717L^ neurons compared to the co-culture model of the control astrocytes and the control neurons was confirmed (Fig. 8f). The GFAP/S100β ratio was increased in the crossed co-culture model (Fig. 8g). In addition, there was an enlargement of the soma in some of the control astrocytes when crossed co-cultured with the *APP* ^V717L^ neurons (Fig. 8e). Thus, this indicated that an astrogliosis-like phenotype can be induced by secreted Aβ from neurons, which is hindered in astrocytic monocultures. These data suggested that our novel co-culture system could expose astrocytic phenotypes of AD with *APP* ^V717L^ mutation. 

## Discussion

This study demonstrated that the co-culture model of iPSC-derived neurons and astrocytes enhanced the maturation of both cell types. Because the maturation of the differentiated cells from iPSCs is often insufficient, the insufficient maturation has been one of the main problems in using iPSC-based models to study of neurological diseases [9]. In our study, we observed significant changes in both neurons and astrocytes differentiated from the iPSCs after co-culture. The morphologies of astrocytes in the co-culture model were different from those in the monoculture model alone [20], reminiscent of those of astrocytes in vivo [31]. The increase in the GFAP/S100β ratio in the co-culture model indicated the increased astrocyte activation [32].

For neurons, both the number of synapses and the number of presynaptic vesicles per synapse were increased in the co-culture model compared to the monoculture model. The increased number of presynaptic vesicles indicated an increased maturation of the neurons [33, 34]. Transcriptomic analyses of the co-culture model of neurons and astrocytes induced from the iPSCs-derived cortical progenitor pool indicated that astrocytes express extracellular proteins that interact with pre- and postsynaptic proteins and mediate maturation of neurons [35]. These extracellular proteins include ITGAV, EFEMP1, and SIRPA, which were the proteins associated with synaptic development [36–38]. In our co-culture model, iPSC-derived astrocytes may also promote neuronal maturation through similar mechanisms. Other underlying mechanisms may involve the secretion of neurotrophic factors such as glial cell line-derived neurotrophic factor (GDNF) directly from the co-cultured astrocytes [39].

Furthermore, since most of the disease models have been differentiated to only a single cell type of cells [reviewed in 6], many of the disease phenotypes due to the abnormal interaction of multiple cell types are still hindered when using a monoculture model. By utilizing the cellular model that includes mutual interactions between different cell types, analyses of the human cellular models in physiological environments would become possible and more disease phenotypes could be recapitulated. To date, there have been several reports of AD-like phenotypes that could be observed using in vitro 2D models generated from the iPSCs carrying fAD mutations on *APP* and *PSEN*. For example, as shown in Table 1, the iPSC-derived neurons could recapitulate the phenotypes associated with the secretion of Aβ, or pTau in some studies. However, the observed phenotypes are mostly limited to the neurons, with only a few reports on glial cells [7, 8, 17, 40–48].

**Table 1:** Summary table of the AD phenotypes evaluated using the iPSC-derived models of donors with familial AD mutations

Mutations	AD-related phenotypes			
	iPSC-derived cortical neurons	References	iPSC-derived astrocytes	References
APP V717L	↑ Aβ42, ↑ Aβ42/Aβ40	[17]	–	–
	↓ Autophagy, ↓ Mitophagy	[40]	–	–
APP V717I	↑ Aβ42, ↑ Aβ38, ↑ Aβ42/Aβ40 , ↑ total Tau, pTau Ser262	[41]	–	–
	↑ Aβ38/Aβ40, ↑ Aβ42/Aβ40	[42]		
APP V717G	↑ Aβ42, ↑ Aβ42/Aβ40	[43]	–	–
APP V717F	–	–	→ Impaired Aβ intake, → Lipid endocytosis	[48]
APP Swe	↑ total Aβ, → Aβ42/Aβ40	[43]	Impaired Aβ intake, ↓ APP, ↓ Lipid endocytosis	[48]
APP SWE/V717I	↑ Aβ42, ↑ Aβ40	[44]	–	–
APP SWE, A692G	↑ Aβ42, ↑ total Aβ, → Aβ42/Aβ40	[43]	–	–
APP Dp	↑ Aβ40, ↑ pTau Ser231, RAB5+ early endosome enlargement	[8]	–	–
APP E693Δ	↑ Intracellular Aβ oligomers, ↑ ER stress and oxidative stress	[17]	–	–
PSEN1 A246E, N141I	→ Aβ40, ↑ Aβ42/Aβ40	[7]	–	–
PSEN1 A246E	↑ Aβ42/Aβ40	[45]	–	–
	↓ Autophagy, ↑ Lysosomal biogenesis	[46]		
PSEN1 Y115H, int4del, M139V, M146I, R278I	↑ Aβ38/Aβ40, ↑ Aβ42/Aβ40	[42]	–	–
PSEN1 M146V, L116P, M233L, A246E	↑ Aβ42, ↓ total Aβ, ↑ Aβ42/Aβ40	[43]	–	–
PSEN1 V89L, L150P	↑ Aβ42, ↑ Aβ40, ↑ Aβ42/Aβ40 ↑pTau Ser262, Ser396, S202/T205, Thr181, Ser400/Thr403/Ser404	[47]	–	–

Fong et al. (2018) demonstrated that iPSC-derived astrocytes from the donors with APP Swe mutations showed impaired Aβ uptake, decreased APP secretion, and reduced lipid endocytosis [48]. We also observed the astrocytic hypertrophy in the monoculture model of AD astrocytes because APPs are also expressed in astrocytes even at low levels [49, 50]. Another astrocytic phenotype, increased astrogliosis due to fAD mutations, was not observed in the iPSC-derived astrocytic monoculture models [48] (Fig. 8c, d). Therefore, the astrocytes co-cultured with neurons exhibited astrogliosis-like phenotypes possibly due to the exposure to high levels of Aβ secreted by neurons [51].

We also showed that our induced neurons and astrocytes interact with each other through the formation of the tripartite synapse structure. Since the tripartite synapse plays an important role in controlling neuronal functions in both physiological and pathological conditions [52], the human cellular model that can recapitulate this structure will be useful for the future studies of several neural diseases, especially those involving the clearance of excessive amounts of glutamate. For example, in AD, recent studies suggest that a soluble oligomeric Aβ causes excitotoxicity through several mechanisms including the stimulation of presynaptic glutamate release, inhibition of glutamate uptake in astrocytes, and alterations of glutamate receptors at the postsynaptic terminal (prolonged activation of S-NMDARs and AMPARs) [53–56]. In some of the frontotemporal temporal dementia spectrum disorders associated with the Tau mutation A152T is associated with neuronal excitotoxicity due to excessive glutamate release from the presynaptic terminal of neurons [57]. Stimulation of the glutamate uptake by the glutamate transporter (EAAT) on astrocytes alleviated the pathological effects [57]. Our co-culture model, which recapitulates the tripartite synapse structure related to these pathologies, will be a useful tool to study these pathogenesis and drug responses in human cells.

There are some previous studies that demonstrated the changes in cellular morphology and function after co-culturing the neural cells. Some studies utilized murine cells from animal models. For example, Jones et al. (2012) showed that the embryonic rat hippocampal neurons suspended on the layer of rat astrocytes developed more synapses compared to the culture without astrocytes [58]. Another study showed that the co-culturing of cerebellar neurons with astrocytes protected neurons from glutamate toxicity [59]. Luchena et al. (2022) developed the triplet co-culture model of the murine rat neurons, astrocytes, and microglia. Similar to our study, the neurons in this triplet co-culture model developed longer neurite branches and expressed more post-synaptic markers, while the co-cultured astrocytes showed ramified morphology compared to the monocultured astrocytes [60]. Furthermore, microglia in the triplet co-culture model express less pro-inflammatory markers and more anti-inflammatory markers [60]. In addition, co-culture of iPSC-derived neurons and rat astrocytes accelerated the maturation of iPSC-derived neurons [61].

The co-culture models of iPSC-derived neural cells have also been reported in recent studies. These models include the co-culture of iPSC-derived neurons and microglia [62], iPSC-derived neurons and astrocytes [63], iPSC-derived motor neurons and microglia [64], and triplet co-culture of neurons, astrocytes, and microglia [16, 65]. Cell maturation in these culture models accelerated compared to the monoculture models of each cell type alone, and the models have been used to study neurodegenerative diseases, including Parkinson’s and Alzheimer’s disease, specifically the inflammatory responses of microglia and the disease pathologies observed in neurons.

In our study, the co-culture model of the individually differentiated neurons and astrocytes newly confirmed the utility of co-culturing the iPSC-derived cells together. In particular, while Guttikonda et al. (2021) used dual SMAD inhibition when inducing neurons as in our study, the astrocytes were derived from the direct induction of human pluripotent stem cells (hPSCs) via transient expression of NFIA [66]. In the triplet co-culture model of Bassil et al. (2021), human primary astrocytes were utilized. In our study, the astrocytes were differentiated from iPSCs via the formation of embryoid bodies and neurospheres, recapitulating the developmental process of astrocytes. Furthermore, the neurons and astrocytes were differentiated from the identical iPSCs prior to co-culture, which demonstrated the alternative method for establishing the co-culture model of human cells. In addition, the serum-free induction method allowed us to observe not only the enhanced maturation of neurons but also the transition of astrocytes from basal to pathological conditions by co-culture.

Finally, several limitations of our study remain. First, we used only one clone of patient-derived iPSCs and demonstrated the phenotypes of AD with the *APP* ^V717L^ mutation [17]. Second, the control line used in this study is the non-AD 201B7 iPSC line [2]. An iPSC line, in which the *APP* ^V717L^ mutation was corrected by genome editing, would provide a better comparison to the *APP* ^V717L^ mutation in terms of phenotypic analyses. Nevertheless, it is noteworthy that the mutual interactions of the iPSC-derived neural cells could enhance cellular maturation and exposed astrocytic phenotypes. The tripartite synapse structure formed in our culture model served as a useful platform for the studies of disease pathogenesis involving this structure. Our study also provides a future perspective to include multiple cell types to study other pathological conditions: for example, the iPSC-derived microglia to assess the inflammatory responses [16, 67, 68], or the iPSC-derived oligodendrocytes to assess the myelination [69, 70]. Further studies on models that include more cell types are also expected to recapitulate more precise interactions of the cells in a dish.

## Conclusions

This present study demonstrated the establishment of the novel co-culture model of neurons and astrocytes that had been individually differentiated from the identical iPSCs. The co-culture system promoted the maturation of both cell types included in the model. Not only the maturation of individual cells, but also the interaction of these cells was confirmed by the formation of the functional tripartite synapse structure. Furthermore, the co-culture model was able to recapitulate astrocytic AD phenotypes including astrocytic hypertrophy and astrogliosis-like phenotype, which have not been reported in other studies using iPSC-based models before. This co-culture system is a powerful tool to study both physiological and pathological conditions of human neural cells.

### Supplementary Information

**Additional file 1: Supplementary Table 1. **Antibodies used in this study. **Supplementary Table 2.** Primers used in this study.

**Additional file 2: Supplementary Video 1. **Ca imaging using Fluo-8 indicator measurement of the control line iPSC-derived neurons in a co-culture model showed higher neuronal activities compared to a neuronal mono-culture model. **Supplementary Video 2.** 20 μM TFB-TBOA (Excitatory Amino Acid Transporter (EAAT) 1,2 inhibitor) treatment to the co-culture model derived from the control iPSC line resulted in hyperexcitability of the neurons measured by Ca imaging using Fluo-8 indicator.

**Additional file 3: Supplementary Fig. 1. **Generation and characterization of iPSC-derived neurons, astrocytes, and co-culture models of the iPSCs of the familial AD donor with *APP* ^V717L^ mutation (APP2E26 line). **a** Immunocytochemistry images of iPSC-derived neurons at PID 45 stained with neuronal markers (MAP2 and NeuN). Scale bar; 50 μm. **b** Relative proportion of MAP2 and NeuN expression compared to Hoechst of the differentiated neurons (*n* = 4). Bars, mean ± SEM. **c** Immunocytochemistry images of iPSC-derived astrocytes (S100β and GFAP). Scale bar; 100 μm. **d** Relative proportion of S100β compared to Hoechst of the differentiated astrocytes (*n* = 4). Bars, mean ± SEM. **e** Comparison of astrocyte differentiation efficacy between the *APP* ^V717L^ iPSC line and the control line (*n* = 4). Bars, mean ± SEM. ns: non-significant (Unpaired t-test). **f** Glutamate reuptake assay for iPSC-derived astrocytes and iPSCs measured at zero, one, and two hours after adding 150 mg/L L-Glutamate (*n* = 3). Bars, mean ± SEM. ****p < 0.0001 (*APP* ^V717L^ astrocytes vs *APP* ^V717L^ iPSCs) (Two-way ANOVA); *p* = 0.4156 (*APP* ^V717L^ astrocytes vs 201B7 astrocytes). **g** Sholl analysis showing the complexity of the iPSC-derived astrocytes of the co-culture model of control 201B7 line (*n* = 11) and *APP* ^V717L^ line (*n* = 11). Bars, mean ± SEM. ns: non-significant (Two-way ANOVA). **h** Sholl analysis showing the complexity of neurites of the iPSC-derived neurons in the co-culture model of control 201B7 line (*n* = 30) and *APP* ^V717L^ line (*n* = 30) (three independent experiments). Bars, mean ± SEM. ns: non-significant (Two- way ANOVA).

**Additional file 4.**

## Data Availability

The data and materials of the current study are available from the corresponding authors upon request.

## References

[1] Takahashi K, Yamanaka S (2006). Induction of pluripotent stem cells from mouse embryonic and adult fibroblast cultures by defined factors. Cell.

[2] Takahashi K, Tanabe K, Ohnuki M, Narita M, Ichisaka T, Tomoda K, Yamanaka S (2007). Induction of pluripotent stem cells from adult human fibroblasts by defined factors. Cell.

[3] Liu G, David BT, Trawczynski M, Fessler RG (2020). Advances in Pluripotent Stem Cells: History, Mechanisms, Technologies, and Applications. Stem Cell Rev Rep.

[4] Stadtfeld M, Hochedlinger K (2010). Induced pluripotency: history, mechanisms, and applications. Genes Dev.

[5] Okano H, Yamanaka S (2014). iPS cell technologies: significance and applications to CNS regeneration and disease. Mol Brain.

[6] Okano H, Morimoto S (2022). iPSC-based disease modeling and drug discovery in cardinal neurodegenerative disorders. Cell Stem Cell.

[7] Yagi T, Ito D, Okada Y, Akamatsu W, Nihei Y, Yoshizaki T, Yamanaka S, Okano H, Suzuki N (2011). Modeling familial Alzheimer's disease with induced pluripotent stem cells. Hum Mol Genet.

[8] Israel MA, Yuan SH, Bardy C, Reyna SM, Mu Y, Herrera C, Hefferan MP, Van Gorp S, Nazor KL, Boscolo FS, Carson CT, Laurent LC, Marsala M, Gage FH, Remes AM, Koo EH, Goldstein LS (2012). Probing sporadic and familial Alzheimer's disease using induced pluripotent stem cells. Nature.

[9] Doss MX, Sachinidis A (2019). Current Challenges of iPSC-Based Disease Modeling and Therapeutic Implications. Cells.

[10] Giacomelli E, Vahsen BF, Calder EL, Xu Y, Scaber J, Gray E, Dafinca R, Talbot K, Studer L (2022). Human stem cell models of neurodegeneration: From basic science of amyotrophic lateral sclerosis to clinical translation. Cell Stem Cell.

[11] Blanchard JW, Victor MB, Tsai LH (2022). Dissecting the complexities of Alzheimer disease with in vitro models of the human brain. Nat Rev Neurol.

[12] Barak M, Fedorova V, Pospisilova V, Raska J, Vochyanova S, Sedmik J, Hribkova H, Klimova H, Vanova T, Bohaciakova D (2022). Human iPSC-Derived Neural Models for Studying Alzheimer's Disease: from Neural Stem Cells to Cerebral Organoids. Stem Cell Rev Rep.

[13] Shimada H, Sato Y, Sasaki T, Shimozawa A, Imaizumi K, Shindo T, Miyao S, Kiyama K, Kondo T, Shibata S, Ishii S, Kuromitsu J, Aoyagi H, Ito D, Okano H (2022). A next-generation iPSC-derived forebrain organoid model of tauopathy with tau fibrils by AAV-mediated gene transfer. Cell Rep Methods.

[14] Sloan SA, Darmanis S, Huber N, Khan TA, Birey F, Caneda C, Reimer R, Quake SR, Barres BA, Paşca SP (2017). Human Astrocyte Maturation Captured in 3D Cerebral Cortical Spheroids Derived from Pluripotent Stem Cells. Neuron.

[15] Sahlgren Bendtsen KM, Hall VJ (2023). The Breakthroughs and Caveats of Using Human Pluripotent Stem Cells in Modeling Alzheimer's Disease. Cells.

[16] Guttikonda SR, Sikkema L, Tchieu J, Saurat N, Walsh RM, Harschnitz O, Ciceri G, Sneeboer M, Mazutis L, Setty M, Zumbo P, Betel D, de Witte LD, Pe'er D, Studer L (2021). Fully defined human pluripotent stem cell-derived microglia and tri-culture system model C3 production in Alzheimer's disease. Nat Neurosci.

[17] Kondo T, Asai M, Tsukita K, Kutoku Y, Ohsawa Y, Sunada Y, Imamura K, Egawa N, Yahata N, Okita K, Takahashi K, Asaka I, Watanabe A, Kadoya C, Nakano R, Watanabe D, Maruyama K, Hori O, Inoue H (2013). Modeling Alzheimer's disease with iPSCs reveals stress phenotypes associated with intracellular Aβ and differential drug responsiveness. Cell Stem Cell.

[18] CiRA. Center for iPS Cell Research and Application, Kyoto University. Available online: https://www.cira.kyoto-u.ac.jp/e/research/img/protocol/Ff-iPSC-culture_protocol_E_v140311.pdf (accessed on 29 June 2023).

[19] Zhou Z, Kakegawa W, Fujimori K, Sho M, Shimamura R, Supakul S, Yoshimatsu S, Kohyama J, Yuzaki M (2023). Hideyuki Okano bioRxiv.

[20] Supakul S, Hatakeyama Y, Leventoux N, Itsuno M, Numata N, Hiramine H, Morimoto S, Iwata A, Maeda S, Okano H (2023). Urine-derived cells from the aged donor for the 2D/3D modeling of neural cells via iPSCs. Aging Brain.

[21] Leventoux N, Morimoto S, Imaizumi K, Sato Y, Takahashi S, Mashima K, Ishikawa M, Sonn I, Kondo T, Watanabe H, Okano H (2020). Human Astrocytes Model Derived from Induced Pluripotent Stem Cells. Cells.

[22] Okada Y, Matsumoto A, Shimazaki T, Enoki R, Koizumi A, Ishii S, Itoyama Y, Sobue G, Okano H (2008). Spatiotemporal recapitulation of central nervous system development by murine embryonic stem cell-derived neural stem/progenitor cells. Stem Cells.

[23] Bannai H, Niwa F, Sherwood MW, Shrivastava AN, Arizono M, Miyamoto A, Sugiura K, Lévi S, Triller A, Mikoshiba K (2015). Bidirectional Control of Synaptic GABAAR Clustering by Glutamate and Calcium. Cell Rep.

[24] Shibata S, Murota Y, Nishimoto Y, Yoshimura M, Nagai T, Okano H, Siomi MC (2015). Immuno-electron microscopy and electron microscopic in situ hybridization for visualizing piRNA biogenesis bodies in Drosophila ovaries. Methods Mol Biol.

[25] Shibata S, Iseda T, Mitsuhashi T, Oka A, Shindo T, Moritoki N, Nagai T, Otsubo S, Inoue T, Sasaki E, Akazawa C, Takahashi T, Schalek R, Lichtman JW, Okano H (2019). Large-area fluorescence and electron microscopic correlative imaging with multibeam scanning electron microscopy. Front Neural Circ.

[26] Ye ZC, Sontheimer H (1998). Astrocytes protect neurons from neurotoxic injury by serum glutamate. Glia.

[27] Perea G, Navarrete M, Araque A (2009). Tripartite synapses: astrocytes process and control synaptic information. Trends Neurosci.

[28] Supakul S, Okano H, Maeda S (2021). Utilization of Human Induced Pluripotent Stem Cells-Derived *In vitro* Models for the Future Study of Sex Differences in Alzheimer's Disease. Front Aging Neurosci.

[29] Murrell JR, Hake AM, Quaid KA, Farlow MR, Ghetti B (2000). Early-onset Alzheimer's disease caused by a new mutation (V717L) in the amyloid precursor protein gene. Arch Neurol.

[30] Dossi E, Vasile F, Rouach N (2018). Human astrocytes in the diseased brain. Brain Res Bull.

[31] Oberheim NA, Takano T, Han X, He W, Lin JH, Wang F, Xu Q, Wyatt JD, Pilcher W, Ojemann JG, Ransom BR, Goldman SA, Nedergaard M (2009). Uniquely hominid features of adult human astrocytes. J Neurosci.

[32] Escartin C, Galea E, Lakatos A, O'Callaghan JP, Petzold GC, Serrano-Pozo A, Steinhäuser C, Volterra A, Carmignoto G, Agarwal A, Allen NJ, Araque A, Barbeito L, Barzilai A, Bergles DE, Bonvento G, Butt AM, Chen WT, Cohen-Salmon M, Cunningham C, Verkhratsky A (2021). Reactive astrocyte nomenclature, definitions, and future directions. Nat Neurosci.

[33] Blue ME, Parnavelas JG (1983). The formation and maturation of synapses in the visual cortex of the rat II Quantitative analysis. J Neurocytol.

[34] Mohrmann R, Lessmann V, Gottmann K (2003). Developmental maturation of synaptic vesicle cycling as a distinctive feature of central glutamatergic synapses. Neuroscience.

[35] Hedegaard A, Monzón-Sandoval J, Newey SE, Whiteley ES, Webber C, Akerman CJ (2020). Pro-maturational Effects of Human iPSC-Derived Cortical Astrocytes upon iPSC-Derived Cortical Neurons. Stem Cell Reports.

[36] Park YK, Goda Y (2016). Integrins in synapse regulation. Nat Rev Neurosci.

[37] Vukovic J, Ruitenberg MJ, Roet K, Franssen E, Arulpragasam A, Sasaki T, Verhaagen J, Harvey AR, Busfield SJ, Plant GW (2009). The glycoprotein fibulin-3 regulates morphology and motility of olfactory ensheathing cells in vitro. Glia.

[38] Wang XX, Pfenninger KH (2006). Functional analysis of SIRPalpha in the growth cone. J Cell Sci.

[39] Cortés D, Carballo-Molina OA, Castellanos-Montiel MJ, Velasco I (2017). The Non-Survival Effects of Glial Cell Line-Derived Neurotrophic Factor on Neural Cells. Front Mol Neurosci.

[40] Fang EF, Hou Y, Palikaras K, Adriaanse BA, Kerr JS, Yang B, Lautrup S, Hasan-Olive MM, Caponio D, Dan X (2019). Mitophagy inhibits amyloid-β and tau pathology and reverses cognitive deficits in models of Alzheimer’s disease. Nat Neurosci.

[41] Muratore CR, Rice HC, Srikanth P, Callahan DG, Shin T, Benjamin LNP, Walsh DM, Selkoe DJ, Young-Pearse TL (2014). The familial Alzheimer’s disease APPV717I mutation alters APP processing and Tau expression in iPSC-derived neurons. Hum Mol Genet.

[42] Arber C, Toombs J, Lovejoy C, Ryan NS, Paterson RW, Willumsen N, Gkanatsiou E, Portelius E, Blennow K, Heslegrave A (2020). Familial Alzheimer’s disease patient-derived neurons reveal distinct mutation-specific effects on amyloid beta. Mol Psychiatry.

[43] Kwart D, Gregg A, Scheckel C, Murphy EA, Paquet D, Duffield M, Fak J, Olsen O, Darnell R, Tessier-Lavigne MA (2019). large panel of isogenic APP and PSEN1 mutant human iPSC neurons reveals shared endosomal abnormalities mediated by APP beta-CTFs, not A beta. Neuron.

[44] Park J, Wetzel I, Marriott I, Dréau D, D’Avanzo C, Kim DY, Tanzi RE, Cho H (2018). A 3D human triculture system modeling neurodegeneration and neuroinflammation in Alzheimer’s disease. Nat Neurosci.

[45] Mahairaki V, Ryu J, Peters A, Chang Q, Li T, Park TS, Burridge PW, Talbot CC, Asnaghi L, Martin LJ (2014). Induced pluripotent stem cells from familial Alzheimer’s disease patients differentiate into mature neurons with amyloidogenic properties. Stem Cells Dev.

[46] Martín-Maestro P, Gargini R, Sproul AA, García E, Antón LC, Noggle S, Arancio O, Avila J, García-Escudero V (2017). Mitophagy failure in fibroblasts and iPSC-derived neurons of Alzheimer’s disease-associated presenilin 1 mutation. Front Mol Neurosci.

[47] Ochalek A, Mihalik B, Avci HX, Chandrasekaran A, Téglási A, Bock I, Giudice ML, Táncos Z, Molnár K, László L (2017). Neurons derived from sporadic Alzheimer’s disease iPSCs reveal elevated TAU hyperphosphorylation, increased amyloid levels, and GSK3B activation. Alzheimer's Res Ther.

[48] Fong LK, Yang M, Chaves RDS, Reyna SM, Langness VF, Woodruff G, Roberts EA, Young JE, Goldstein LSB (2018). Full-length amyloid precursor protein regulates lipoprotein metabolism and amyloid-beta clearance in human astrocytes. J Biol Chem.

[49] Golde TE, Estus S, Usiak M, Younkin LH, Younkin SG (1990). Expression of beta amyloid protein precursor mRNAs: recognition of a novel alternatively spliced form and quantitation in Alzheimer's disease using PCR. Neuron.

[50] Frost GR, Li YM (2017). The role of astrocytes in amyloid production and Alzheimer's disease. Open Biol.

[51] Olsen M, Aguilar X, Sehlin D, Fang XT, Antoni G, Erlandsson A, Syvänen S (2018). Astroglial Responses to Amyloid-Beta Progression in a Mouse Model of Alzheimer's Disease. Mol Imag Biol.

[52] Halassa MM, Fellin T, Haydon PG (2007). The tripartite synapse: roles for gliotransmission in health and disease. Trends Mol Med.

[53] Mucke L, Masliah E, Yu GQ, Mallory M, Rockenstein EM, Tatsuno G (2000). High-level neuronal expression of abeta 1–42 in wild-type human amyloid protein precursor transgenic mice: synaptotoxicity without plaque formation. J Neurosci.

[54] Li S, Hong S, Shepardson NE, Walsh DM, Shankar GM, Selkoe D (2009). Soluble oligomers of amyloid Beta protein facilitate hippocampal long-term depression by disrupting neuronal glutamate uptake. Neuron.

[55] Shankar GM, Bloodgood BL, Townsend M, Walsh DM, Selkoe DJ, Sabatini BL (2007). Natural oligomers of the Alzheimer amyloid-beta protein induce reversible synapse loss by modulating an NMDA-type glutamate receptor-dependent signaling pathway. J Neurosci.

[56] Rudy CC, Hunsberger HC, Weitzner DS, Reed MN (2015). The role of the tripartite glutamatergic synapse in the pathophysiology of Alzheimer's disease. Aging Dis.

[57] Decker JM, Krüger L, Sydow A, Dennissen FJ, Siskova Z, Mandelkow E, Mandelkow EM (2016). The Tau/A152T mutation, a risk factor for frontotemporal-spectrum disorders, leads to NR2B receptor-mediated excitotoxicity. EMBO Rep.

[58] Jones EV, Cook D, Murai KK (2012). A neuron-astrocyte co-culture system to investigate astrocyte-secreted factors in mouse neuronal development. Methods Mol Biol.

[59] Brown DR (1999). Neurons depend on astrocytes in a coculture system for protection from glutamate toxicity. Mol Cell Neurosci.

[60] Luchena C, Zuazo-Ibarra J, Valero J, Matute C, Alberdi E, Capetillo-Zarate E (2022). A Neuron, Microglia, and Astrocyte Triple Co-culture Model to Study Alzheimer's Disease. Front Aging Neurosci.

[61] Meyer K, Feldman HM, Lu T, Drake D, Lim ET, Ling KH, Bishop NA, Pan Y, Seo J, Lin YT, Su SC, Church GM, Tsai LH, Yankner BA (2019). REST and Neural Gene Network Dysregulation in iPSC Models of Alzheimer's Disease. Cell Rep.

[62] Haenseler W, Sansom SN, Buchrieser J, Newey SE, Moore CS, Nicholls FJ, Chintawar S, Schnell C, Antel JP, Allen ND, Cader MZ, Wade-Martins R, James WS, Cowley SA (2017). A Highly Efficient Human Pluripotent Stem Cell Microglia Model Displays a Neuronal-Co-culture-Specific Expression Profile and Inflammatory Response. Stem cell reports.

[63] di Domenico A, Carola G, Calatayud C, Pons-Espinal M, Muñoz JP, Richaud-Patin Y, Fernandez-Carasa I, Gut M, Faella A, Parameswaran J, Soriano J, Ferrer I, Tolosa E, Zorzano A, Cuervo AM, Raya A, Consiglio A (2019). Patient-Specific iPSC-Derived Astrocytes Contribute to Non-Cell-Autonomous Neurodegeneration in Parkinson's Disease. Stem Cell Reports.

[64] Vahsen BF, Gray E, Candalija A, Cramb KML, Scaber J, Dafinca R, Katsikoudi A, Xu Y, Farrimond L, Wade-Martins R, James WS, Turner MR, Cowley SA, Talbot K (2022). Human iPSC co-culture model to investigate the interaction between microglia and motor neurons. Sci Rep.

[65] Bassil R, Shields K, Granger K, Zein I, Ng S, Chih B (2021). Improved modeling of human AD with an automated culturing platform for iPSC neurons, astrocytes and microglia. Nat Commun.

[66] Tchieu J, Calder EL, Guttikonda SR, Gutzwiller EM, Aromolaran KA, Steinbeck JA, Goldstein PA, Studer L (2019). NFIA is a gliogenic switch enabling rapid derivation of functional human astrocytes from pluripotent stem cells. Nat Biotechnol.

[67] Sonn I, Honda-Ozaki F, Yoshimatsu S, Morimoto S, Watanabe H, Okano H (2022). Single transcription factor efficiently leads human induced pluripotent stem cells to functional microglia. Inflamm Regen.

[68] Muzio L, Viotti A, Martino G (2022). Microglia in Neuroinflammation and Neurodegeneration: From Understanding to Therapy. Front Neurosci.

[69] Ehrlich M, Mozafari S, Glatza M, Starost L, Velychko S, Hallmann AL, Cui QL, Schambach A, Kim KP, Bachelin C, Marteyn A, Hargus G, Johnson RM, Antel J, Sterneckert J, Zaehres H, Schöler HR, Baron-Van Evercooren A, Kuhlmann T (2017). Rapid and efficient generation of oligodendrocytes from human induced pluripotent stem cells using transcription factors. Proc Natl Acad Sci USA.

[70] Simons M, Nave KA (2015). Oligodendrocytes: Myelination and Axonal Support. Cold Spring Harb Perspect Biol.

