# Supplementary material for: Mutual interaction of neurons and astrocytes derived from iPSCs with APP V717L mutation developed the astrocytic phenotypes of Alzheimer’s disease
Source: Inflamm Regen. 2024 Feb 28;44:8. doi: 10.1186/s41232-023-00310-5 (PMC10900748; doi:10.1186/s41232-023-00310-5)
Supplement: Supplementary file 1 — Additional file 1: Supplementary Table 1. Antibodies used in this study. Supplementary Table 2. Primers used in this study. [file 41232_2023_310_MOESM1_ESM.docx]

**Supplementary Table 1.** Antibodies used in this study.

| **Antibodies used for immunocytochemistry and immunohistochemistry** | | | |
| --- | --- | --- | --- |
|  | **Antibody** | **Dilution** | **Company Cat # and RRID** |
| Astrocyte marker (ICC) | Rat Anti-GFAP | 1:500 | Millipore Cat# 345860  RRID:AB_2109651 |
| Astrocyte marker (ICC) | Rabbit Anti-EAAT1 | 1:100 | Abcam Cat# ab416  RRID:AB_304334 |
| Astrocyte marker (ICC) | Rabbit Anti-EAAT2 | 1:50 | Abcam Cat# ab41621 RRID:AB_941782 |
| Astrocyte marker (ICC) | Mouse Anti-S100β | 1:250 | Sigma-Aldrich Cat# S2532 RRID:AB_477499 |
| Astrocyte marker (ICC) | Mouse Anti-CD44 | 1:500 | Abcam Cat# ab6124  RRID:AB_305297 |
| Neuronal marker (ICC) | Rabbit Anti-MAP2 | 1:1000 | Millipore Cat# AB5622  RRID:AB_91939 |
| Neuronal marker (ICC) | Rabbit Anti-MAP2 | 1:100 | Cell Signaling Technology Cat# 4542 RRID:AB_10693782 |
| Neuronal marker (ICC) | Mouse Anti-NeuN | 1:200 | Abcam Cat# ab104224  RRID:AB_10711040 |
| Synaptic marker (ICC) | Mouse Anti-NMDAR2B | 1:500 | BD Biosciences Cat# 610417  RRID:AB_397797 |
| Synaptic marker (ICC) | Rabbit Anti-Synapsin1 | 1:2000 | Sigma-Aldrich Cat# S193  RRID:AB_261457 |
| Synaptic marker (ICC) | Rabbit Anti-PSD95 | 1:1000 | Frontier Institute Cat# PSD95-Rb RRID:AB_2571611 |
| Synaptic marker (ICC) | Guinea Pig Anti-VGLUT2 | 1:500 | Synaptic Systems Cat# 135 404 RRID:AB_887884 |
| Fluorescence protein marker (ICC) | Rabbit Anti-RFP | 1:500 | MBL International Cat# PM005  RRID:AB_591279 |
| Secondary Antibody | Alexa Flour 488  Goat Anti-Rabbit IgG | 1:500 | Thermo Fisher Scientific Cat# A11034 RRID:AB_2576217 |
| Secondary Antibody | Alexa Flour 555  Goat Anti-Rabbit IgG | 1:500 | Molecular Probes Cat# A21429  RRID:AB 2535850 |
| Secondary Antibody | Alexa Flour 647  Goat Anti-Rabbit IgG | 1:500 | Molecular Probes Cat# A21245 RRID:AB_141775 |
| Secondary Antibody | Alexa Flour 488  Goat Anti-Mouse IgG | 1:500 | Molecular Probes Cat# A11029  RRID:AB_2534088 |
| Secondary Antibody | Alexa Flour 555  Goat Anti-Mouse IgG | 1:500 | Molecular Probes Cat# A21424  RRID:AB_141780 |
| Secondary Antibody | Alexa Flour 647  Goat Anti-Mouse IgG | 1:500 | Thermo Fisher Scientific Cat# A21236 RRID:AB_2535805 |
| Secondary Antibody | Alexa Flour 488  Goat Anti-Rat IgG | 1:500 | Molecular Probes Cat# A11006 RRID:AB_141373 |
| Secondary Antibody | Alexa Flour 555  Goat Anti-Rat IgG | 1:500 | Molecular Probes Cat# A21434  RRID:AB_141733 |
| Secondary Antibody | Alexa Flour 647  Goat Anti-Rat IgG | 1:500 | Molecular Probes Cat# A21247  RRID:AB_141778 |
| Secondary Antibody | Alexa Flour 647  Goat Anti-Guinea Pig IgG | 1:500 | Molecular Probes Cat# A21450 RRID:AB_141882 |

**Supplementary Table 2** Primers used in this study.

| **Primers** | | |
| --- | --- | --- |
|  | **Target/Product length** | **Forward/Reverse primer (5′-3′)** |
| Differentiation marker (qPCR) | *OCT4*/144bp | F: GACAGGGGGAGGGGAGGAGCTAG R: CTTCCCTCCAACCAGTTGCCCCA |
| Neuronal marker (qPCR) | *FOXG1*/131bp | F: CCCGTCAA TGACTTCGCAGA  R: GTCCCGTCGTAAAACTTGGC |
| Neuronal marker (qPCR) | *PAX6*/109bp | F: ACCACACCGGTTTCCTCCTTCACA  R: TTGCCATGGTGAAGCTGGGCAT |
| Astrocyte marker (qPCR) | *S100β*/134bp | F: GTGGCCCTCATCGACGTTTT  R: ACCTCCTGCTCTTTGATTTCCTCT |
| Astrocyte marker (qPCR) | *GFAP*/93bp | F: TGTGAGGCAGAAGCTCCAGGATGA  R: AGGGTGGCTTCATCTGCTTCCTGT |
| Astrocyte marker (qPCR) | *CD44*/118bp | F: CTCCAGTGAAAGGAGCAGCA  R: AGCAGGGATTCTGTCTGTGC |
| House-Keeping Gene (qPCR) | *ACTB*/155bp | F: TGAAGTGTGACGTGGACATC  R: GGAGGAGCAATGATCTTGAT |
| Targeted AD mutation (DNA sequencing) | *APPV717L*/97bp | F: CCAAATGTCCCCTGCATT R: CTCTCATAGTCTTAATTCCCAC |
