# Supplementary material for: Mutual interaction of neurons and astrocytes derived from iPSCs with APP V717L mutation developed the astrocytic phenotypes of Alzheimer’s disease
Source: Inflamm Regen. 2024 Feb 28;44:8. doi: 10.1186/s41232-023-00310-5 (PMC10900748; doi:10.1186/s41232-023-00310-5)
Supplement: Supplementary file 2 — Additional file 2: Supplementary Video 1. Ca imaging using Fluo-8 indicator measurement of the control line iPSC-derived neurons in a co-culture model showed higher neuronal activities compared to a neuronal mono-culture model. Supplementary Video 2. 20 μM TFB-TBOA (Excitatory Amino Acid Transporter (EAAT) 1,2 inhibitor) treatment to the co-culture model derived from the control iPSC line resulted in hyperexcitability of the neurons measured by Ca imaging using Fluo-8 indicator. [file 41232_2023_310_MOESM2_ESM.pptx]

## Slide 1
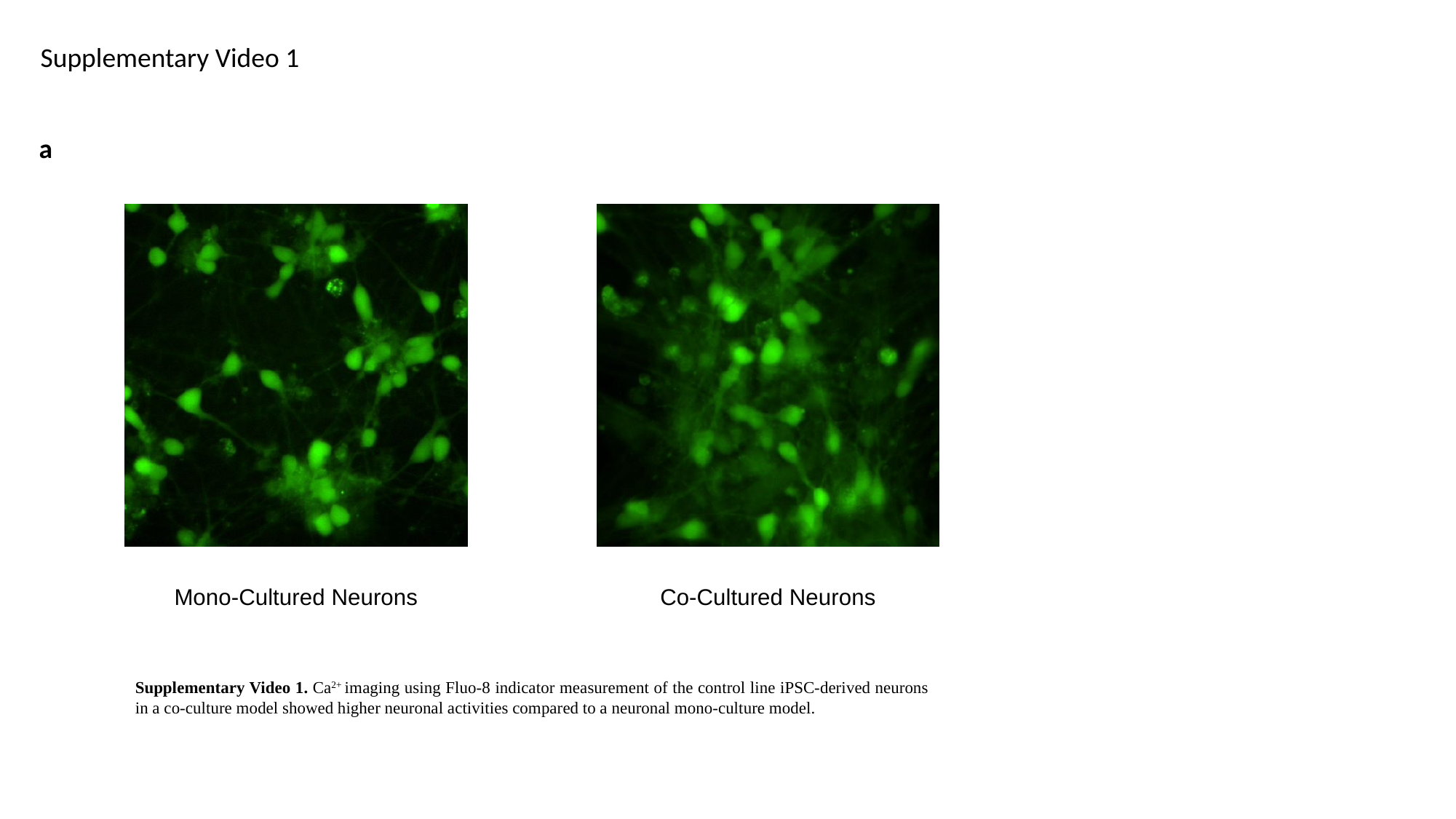

Supplementary Video 1
a
Mono-Cultured Neurons
Co-Cultured Neurons
Supplementary Video 1. Ca2+ imaging using Fluo-8 indicator measurement of the control line iPSC-derived neurons in a co-culture model showed higher neuronal activities compared to a neuronal mono-culture model.

## Slide 2
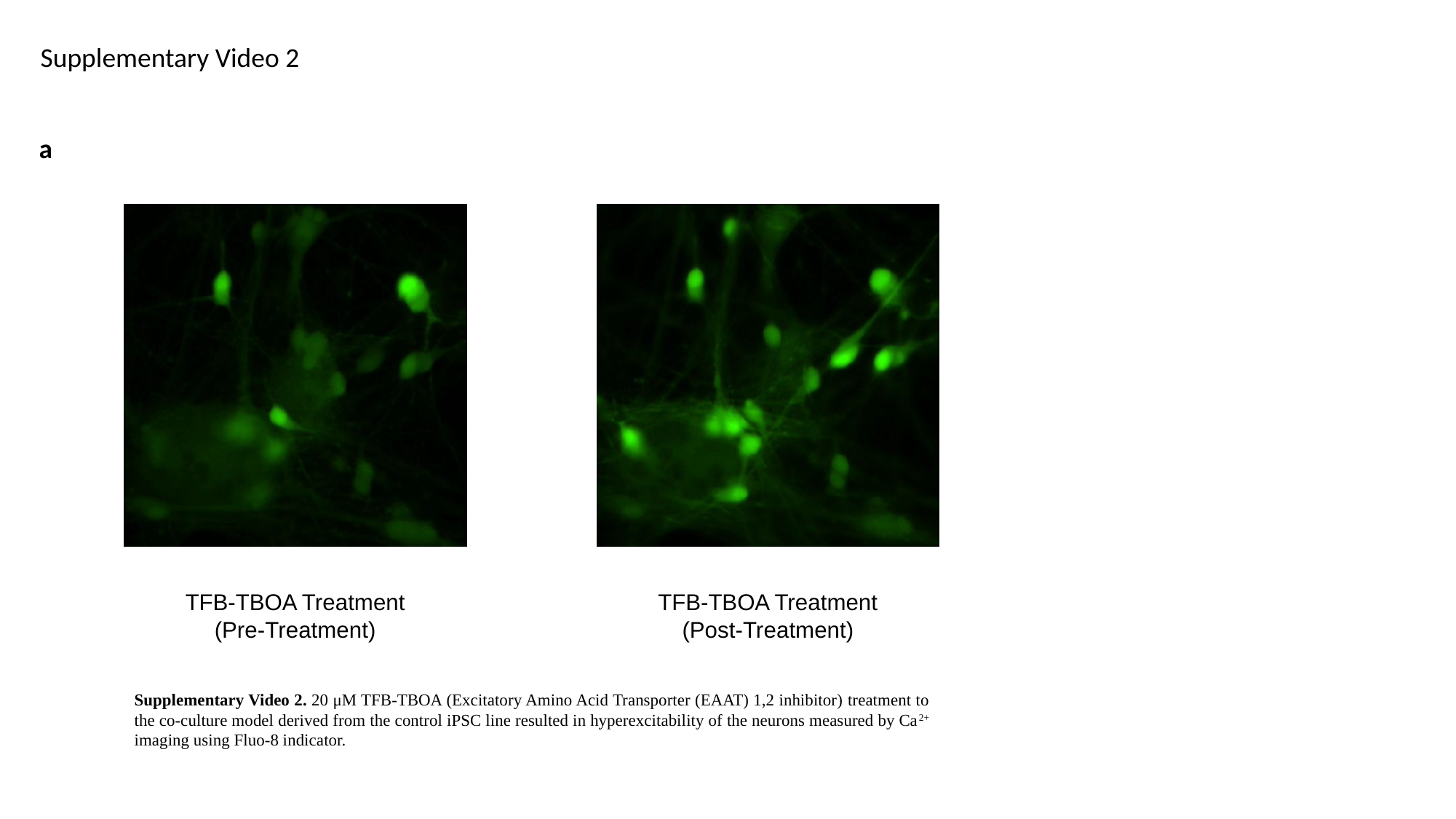

Supplementary Video 2
a
TFB-TBOA Treatment
(Pre-Treatment)
TFB-TBOA Treatment
(Post-Treatment)
Supplementary Video 2. 20 μM TFB-TBOA (Excitatory Amino Acid Transporter (EAAT) 1,2 inhibitor) treatment to the co-culture model derived from the control iPSC line resulted in hyperexcitability of the neurons measured by Ca2+ imaging using Fluo-8 indicator.
