# Supplementary material for: Mutual interaction of neurons and astrocytes derived from iPSCs with APP V717L mutation developed the astrocytic phenotypes of Alzheimer’s disease
Source: Inflamm Regen. 2024 Feb 28;44:8. doi: 10.1186/s41232-023-00310-5 (PMC10900748; doi:10.1186/s41232-023-00310-5)
Supplement: Supplementary file 3 — Additional file 3: Supplementary Fig. 1. Generation and characterization of iPSC-derived neurons, astrocytes, and co-culture models of the iPSCs of the familial AD donor with APP V717L mutation (APP2E26 line). a Immunocytochemistry images of iPSC-derived neurons at PID 45 stained with neuronal markers (MAP2 and NeuN). Scale bar; 50 μm. b Relative proportion of MAP2 and NeuN expression compared to Hoechst of the differentiated neurons (n = 4). Bars, mean ± SEM. c Immunocytochemistry images of iPSC-derived astrocytes (S100β and GFAP). Scale bar; 100 μm. d Relative proportion of S100β compared to Hoechst of the differentiated astrocytes (n = 4). Bars, mean ± SEM. e Comparison of astrocyte differentiation efficacy between the APP V717L iPSC line and the control line (n = 4). Bars, mean ± SEM. ns: non-significant (Unpaired t-test). f Glutamate reuptake assay for iPSC-derived astrocytes and iPSCs measured at zero, one, and two hours after adding 150 mg/L L-Glutamate (n = 3). Bars, mean ± SEM. ****p < 0.0001 (APP V717L astrocytes vs APP V717L iPSCs) (Two-way ANOVA); p = 0.4156 (APP V717L astrocytes vs 201B7 astrocytes). g Sholl analysis showing the complexity of the iPSC-derived astrocytes of the co-culture model of control 201B7 line (n = 11) and APP V717L line (n = 11). Bars, mean ± SEM. ns: non-significant (Two-way ANOVA). h Sholl analysis showing the complexity of neurites of the iPSC-derived neurons in the co-culture model of control 201B7 line (n = 30) and APP V717L line (n = 30) (three independent experiments). Bars, mean ± SEM. ns: non-significant (Two- way ANOVA). [file 41232_2023_310_MOESM3_ESM.docx]

**Supplementary Fig 1** Generation and characterization of iPSC-derived neurons, astrocytes, and co-culture models of the iPSCs of the familial AD donor with *APP* ^V717L^ mutation (APP2E26 line). **a** Immunocytochemistry images of iPSC-derived neurons at PID 45 stained with neuronal markers (MAP2 and NeuN). Scale bar; 50 μm. **b** Relative proportion of MAP2 and NeuN expression compared to Hoechst of the differentiated neurons (n = 4). Bars, mean ± SEM. **c** Immunocytochemistry images of iPSC-derived astrocytes (S100β and GFAP). Scale bar; 100 μm. **d** Relative proportion of S100β compared to Hoechst of the differentiated astrocytes (n = 4). Bars, mean ± SEM. **e** Comparison of astrocyte differentiation efficacy between the *APP* ^V717L^ iPSC line and the control line (n = 4). Bars, mean ± SEM. ns: non-significant (Unpaired t-test). **f** Glutamate reuptake assay for iPSC-derived astrocytes and iPSCs measured at zero, one, and two hours after adding 150 mg/L L-Glutamate (n = 3). Bars, mean ± SEM. *****p* < 0.0001 (*APP* ^V717L^ astrocytes vs *APP* ^V717L^ iPSCs) (Two-way ANOVA); *p* = 0.4156 (*APP* ^V717L^ astrocytes vs 201B7 astrocytes). **g** Sholl analysis showing the complexity of the iPSC-derived astrocytes of the co-culture model of control 201B7 line (n = 11) and *APP* ^V717L^ line (n = 11). Bars, mean ± SEM. ns: non-significant (Two-way ANOVA). **h** Sholl analysis showing the complexity of neurites of the iPSC-derived neurons in the co-culture model of control 201B7 line (n = 30) and *APP* ^V717L^ line (n = 30) (three independent experiments). Bars, mean ± SEM. ns: non-significant (Two-way ANOVA).
